# Patterns of seasonality and group membership characterize the gut microbiota in a longitudinal study of wild Verreaux's sifakas (*Propithecus verreauxi*)

**DOI:** 10.1002/ece3.3148

**Published:** 2017-06-15

**Authors:** Andrea Springer, Claudia Fichtel, Gabriel A. Al‐Ghalith, Flávia Koch, Katherine R. Amato, Jonathan B. Clayton, Dan Knights, Peter M. Kappeler

**Affiliations:** ^1^ Behavioral Ecology and Sociobiology Unit German Primate Center Göttingen Germany; ^2^ Institute for Parasitology, Centre for Infection Medicine University of Veterinary Medicine Hannover Hanover Germany; ^3^ Biomedical Informatics and Computational Biology University of Minnesota Minneapolis MN USA; ^4^ Department of Psychology University of Lethbridge Lethbridge AB Canada; ^5^ Department of Anthropology Northwestern University Evanston IL USA; ^6^ Department of Veterinary and Biomedical Sciences University of Minnesota Saint Paul MN USA; ^7^ GreenViet Biodiversity Conservation Center Son Tra District, Danang Vietnam; ^8^ Biotechnology Institute University of Minnesota Saint Paul MN USA; ^9^ Computer Science and Engineering University of Minnesota Minneapolis MN USA; ^10^ Department of Sociobiology/Anthropology University of Göttingen Göttingen Germany; ^11^ Present address: Institute for Parasitology Centre for Infection Medicine University of Veterinary Medicine Hannover Hanover Germany

**Keywords:** diet, Firmicutes‐Bacteroidetes ratio, folivory, intestinal microbiota, *Propithecus*, seasonality

## Abstract

The intestinal microbiota plays a major role in host development, metabolism, and health. To date, few longitudinal studies have investigated the causes and consequences of microbiota variation in wildlife, although such studies provide a comparative context for interpreting the adaptive significance of findings from studies on humans or captive animals. Here, we investigate the impact of seasonality, diet, group membership, sex, age, and reproductive state on gut microbiota composition in a wild population of group‐living, frugi‐folivorous primates, Verreaux's sifakas (*Propithecus verreauxi*). We repeatedly sampled 32 individually recognizable animals from eight adjacent groups over the course of two different climatic seasons. We used high‐throughput sequencing of the 16S rRNA gene to determine the microbiota composition of 187 fecal samples. We demonstrate a clear pattern of seasonal variation in the intestinal microbiota, especially affecting the Firmicutes‐Bacteroidetes ratio, which may be driven by seasonal differences in diet. The relative abundances of certain polysaccharide‐fermenting taxa, for example, Lachnospiraceae, were correlated with fruit and fiber consumption. Additionally, group membership influenced microbiota composition independent of season, but further studies are needed to determine whether this pattern is driven by group divergences in diet, social contacts, or genetic factors. In accordance with findings in other wild mammals and primates with seasonally fluctuating food availability, we demonstrate seasonal variation in the microbiota of wild Verreaux's sifakas, which may be driven by food availability. This study adds to mounting evidence that variation in the intestinal microbiota may play an important role in the ability of primates to cope with seasonal variation in food availability.

## 1 INTRODUCTION

Over recent years, evidence has accumulated that the gut microbial community of vertebrates is shaped by physiological, dietary, and social influences (Amato et al., 2013; Koren et al., 2012; Moeller et al., 2016; Tung et al., 2015; Turnbaugh, Bäckhed, Fulton, & Gordon, 2008). The microbiota in turn affects host development, metabolism, and health (Clemente, Ursell, Parfrey, & Knight, 2012; Morgan et al., 2012; Sommer & Bäckhed, 2013; Sommer et al., 2016). Host–microbiota interactions are, thus, an important factor in vertebrate ecology and evolution (Amato, 2016). However, in contrast to numerous studies on humans and laboratory animals, few studies have investigated the causes and consequences of microbiota variation in wild vertebrates, and studies based on repeated sampling of individually recognizable animals are especially rare (but see Aivelo, Laakkonen, & Jernvall, 2016; Amato et al., 2014, 2015).

Diet is one of the principal factors shaping the composition of the gut microbiota. While the presence of approximately 60% of bacterial species, and even specific strains, can be remarkably stable (in humans; Faith et al., 2013), short‐term changes in diet can promptly affect relative bacterial abundances (Amato et al., 2015; David et al., 2014; Williams et al., 2013). For example, switching from a high‐fat/low‐fiber to a low‐fat/high‐fiber diet affects the relative abundances of several microbial taxa in humans, for example, *Bacteroides* and *Ruminococcus*, within 24 hrs (David et al., 2014; Wu et al., 2011). These diet‐related changes may increase energy extraction from food and consequently alter host metabolic pathways (Sommer & Bäckhed, 2013; Turnbaugh et al., 2006).

In wildlife, naturally occurring changes in food availability often cause seasonal shifts in energy intake and diet composition (e.g., Norscia, Carrai, & Borgognini‐Tarli, 2006; Wrangham, Conklin‐Brittain, & Hunt, 1998) and may, consequently, also affect the gut microbiota. For example, western lowland gorillas (*Gorilla gorilla*) display an increase in the abundance of microbes involved in fiber breakdown in response to low fruit availability (Gomez et al. 2016). In black howler monkeys (*Alouatta pigra*), an increase in the abundance of Ruminococcaceae, which are efficient fermenters of nonsoluble carbohydrates, was noted during periods of reduced energy intake and might be a mechanism to compensate for low food quality (Amato et al., 2015). However, this hypothesis has not been tested on other species experiencing similar seasonal variation in diet.

Furthermore, host development and increased metabolic demands (e.g., during growth or reproduction) may be associated with distinct changes in the gut microbiota. In humans, the gut microbiota becomes more diverse from infancy to adulthood, although the opposite has been reported for chimpanzees (*Pan troglodytes*) (Degnan et al., 2012). Distinct changes in the gut microbiota also occur at certain life events, such as weaning (Koenig et al., 2011; McKenney, Rodrigo, & Yoder, 2015).

Additionally, characteristic sex differences in microbiota composition, potentially related to endocrine/steroid differences, have been found in humans (Dominianni et al., 2015) as well as in nonhuman primates (Amato et al., 2014). Specifically, females showed lower abundances of Bacteroidetes relative to Firmicutes than males. Furthermore, women experience profound changes in the gut microbiota during pregnancy, potentially adapting the metabolism to an increased energetic demand (Koren et al., 2012).

Finally, microbial assemblages may in part be hereditary, as host genotype may influence susceptibility to colonization by certain microbes (Bonder et al., 2016; Kovacs et al., 2011; van Opstal & Bordenstein, 2015; Turpin et al., 2016). However, it remains unclear to which extent host relatedness contributes to microbiota similarity in wildlife, especially in relation to the factors discussed above. Several studies on wild primates suggested that environmental factors have a bigger impact on the gut microbiota than genotype (Degnan et al., 2012; Moeller & Ochman, 2013), but further studies on other species are needed.

In this study, we characterize seasonal variation in the gut microbiota of a wild population of group‐living, frugi‐folivorous lemurs, Verreaux's sifakas (*Propithecus verreauxi*). Specifically, we investigate the influence of diet, age, sex, reproductive state, and membership in each of eight adjacent social groups on gut microbiota composition. Seasonal as well as interindividual differences in gut microbiota composition have been detected in wild Verreaux's sifakas (Fogel, 2015), but the underlying host traits and nutritional correlates have not been investigated.

Here, we predicted that the composition of intestinal microbiota in wild Verreaux's sifakas should vary in accordance with variation in diet. In the dry deciduous forests in western Madagascar, Verreaux's sifakas experience pronounced seasonality: A dry, lean season, characterized by the loss of foliage in many tree species, lasts from May to October, followed by a wet season from November to March. During the dry season, Verreaux's sifakas display a significant decrease in body mass and body fat (Lewis & Kappeler, 2005), indicating a seasonal shortage of energy intake relative to energy expenditure. Indeed, they rely heavily on fruits during the wet season, while a dietary shift to mostly mature leaves, which tend to be low in energy, occurs in the dry season (Koch et al., 2017; Norscia et al., 2006). We predicted that a higher intake of leaves relative to fruit would translate into greater abundances of fiber‐degrading bacteria.

Additionally, we predicted that host age and sex would influence gut microbiota composition, and that the microbiota of lactating and pregnant females would differ from that of males. Reproduction is seasonal, with a short mating period in January/February followed by 5 months of gestation, so that females lactate throughout the dry season and weaning coincides with peak food availability (Koch et al., 2017). During the late stage of lactation, females have a higher nutrient intake than males (Koch et al., 2017). Furthermore, peri‐parturient females increase their consumption of tannins (Carrai, Borgognini‐Tarli, Huffman, & Bardi, 2003). Thus, physiological differences as well as dietary divergence might contribute to sex differences in gut microbiota composition.

Finally, the study population comprised eight neighboring, multimale multifemale groups. Given that these groups inhabit distinct territories with limited overlap and potentially divergent food availability, and that greater genetic relatedness exists within than between groups (Kappeler & Fichtel, 2012), we expected microbiota composition to vary between groups.

## 2 MATERIALS AND METHODS

**Figure 1:**
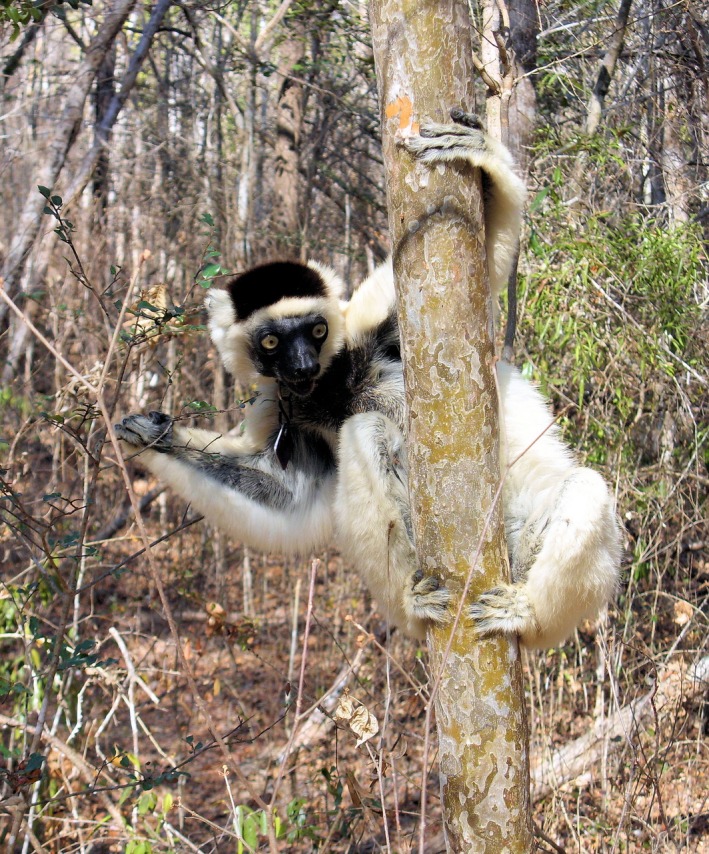
Collared Verreaux's sifaka (*Propithecus verreauxi*) feeding on leaves in Kirindy Forest, Madagascar

The study was carried out in Kirindy Forest, western Madagascar (approx. 44°39′E, 20°03′S). The 90‐ha study area is part of a field station operated by the German Primate Center within a forestry concession managed by Centre National de Formation, d'Etudes et de Recherche en Environnement et Foresterie (CNFEREF). As part of a long‐term study (Kappeler & Fichtel, 2012), Kirindy sifakas have been habituated to human observers and individually marked with unique collars (Figure 1). All necessary research permits were obtained from the respective authorities (Ministère des Eaux et Forêts of Madagascar; Commission ad hoc Flore et Faune of Madagascar; CNFEREF; The Federal Agency for Nature Conservation of Germany).

### 2.1 Fecal sample collection, DNA isolation, and sequencing

We collected fecal samples from 32 individuals (five juveniles (1–4 years of age), 12 adult females and 15 adult males (5–21 years)), representing eight social groups with adjacent, partially overlapping home ranges. Group size ranged from 3 to 7 individuals, with 1–3 adult females, 1–3 adult males, and up to two juveniles per group. One sample per individual per month was collected during two periods, August–October 2013 (late dry season, 96 samples) and March–May 2014 (late wet season/early dry season, 91 samples). During the first sampling period, seven females were lactating, while eight females were known to be pregnant during the second period. Samples were collected from the ground within 2 min of observed defecation. All fecal samples were stored in RNA later at −20°C until analysis.

DNA was extracted using the PowerSoil DNA isolation kit (MoBio, Carlsbad, CA). We used a two‐step PCR protocol to reduce experimental errors and improve accuracy (Gohl et al., 2016). After qPCR‐amplification with KAPA HiFi polymerase (Kapa Biosystems, Woburn, MA), the V4 region of bacterial 16S rRNA was amplified using primers 515F/806R (Supplementary Methods). Amplicons were barcoded, pooled in equal concentrations and denatured with NaOH, diluted to 8 pmol/L in Illumina's HT1 buffer, and spiked with 15% PhiX. Next‐generation sequencing was performed with the Illumina MiSeq 600 cycle v3 kit.

### 2.2 Sequence quality control, operational taxonomic unit (OTU) picking, and taxonomic assignment

After trimming of Nextera adaptors using Trimmomatic (Bolger, Lohse, & Usadel, 2014), we used FLASH read stitching (Magoc & Salzberg, 2011) to generate uniform, full‐length consensus V4 reads. Stitching parameters enforced overlap between 285 and 300 bp, to remove any reads still contaminated with adaptors; reads failing to stitch were discarded. On average, 93.4% of reads were stitched successfully per sample (*SD*: 6%, see also Fig. S1). Reads were quality‐trimmed using an in‐house script before and after five consecutive bases with Phred score ≥20. This resulted in a total of 6,154,792 reads (mean 32,565 per sample).

We performed closed‐reference OTU‐picking and assigned taxonomy using the NINJA‐OPS classifier (Al‐Ghalith, Montassier, Ward, & Knights, 2016) with an 87% similarity threshold against the Greengenes 94% representative sequence database. We used the 87% threshold, which is the minimum generalized threshold for family‐level taxonomy discrimination (Yarza et al., 2014), because despite the high quality of the sequences as validated by quality scores, read lengths, and BLAST tests on a randomized subset of 1,000 reads, only approx. 5% of reads showed high‐identity matches (≥0.97 end‐to‐end) to known sequences (for details on sequence quality assessment as well as a discussion regarding the possible reasons of low read mapping, see the Supplementary Methods Discussion). Therefore, we assigned taxonomy on a per‐read basis, instead of performing initial OTU clustering followed by taxonomic profiling of the resulting clusters. The 94% representative sequence database was used to prevent overspecific assignment finer than the genus level. This resulted in 88.5% of sequences mapping to known taxonomies. This dataset was rarefied to 8,000 reads per sample before analysis and used for all analyses except genus‐level investigations. Rarefaction curves were inspected to verify that the level of rarefaction adequately captured the phylogenetic diversity within all samples (Fig. S3).

To investigate monthly variation within known taxa on the genus level, we used NINJA‐OPS to pick OTUs against the 97% Greengenes (V13.8) representative sequence database with a 94% similarity threshold, which resulted in approx. 34% of sequences mapping to known genera. This dataset was rarefied to 2,000 reads per sample before analysis.

### 2.3 Nutritional data collection

Data on diet composition and samples for nutritional analyses were collected from April 2012 to April 2013, the year prior to fecal sampling. However, group composition showed only minor differences between both study years. Focal animal observations were carried out on 18 adult individuals (nine females, nine males), during each month of the year, resulting in 1,064 hrs of observation (mean 6 hr ± 3 per focal individual) (see Koch et al. (2017) for details). These 18 individuals represented all eight study groups, and 15 of them still belonged to the same group during the period of fecal sample collection.

During observations, the food type (young or mature leaves, fruits, flowers) and plant species consumed by the focal individual were recorded. For all food resources eaten by a focal individual consecutively for more than 5 min, intake was estimated by multiplying bite rate by the estimated dry weight of the specific matter ingested per bite (as described in detail in Koch et al. (2017)).

Samples of food items were processed, shipped to Germany and analyzed at the University of Hamburg as described in Koch et al. (2017). Overall energy intake was estimated based on the conventional conversion values of 4 kcal per gram protein, 4 kcal per gram of nonstructural carbohydrates, and 9 kcal per gram of fat (Committee on Animal Nutrition, 2003). The value for fiber was 1.2 kcal per gram, based on a study that investigated fiber digestibility in sifakas in captivity (Campbell, Eisemann, Glander, & Crissey, 1999). Leaves were not analyzed for “fat” because ether extracts from leaves are very low.

Additionally, we recorded monthly phenology of 692 trees throughout both study years (March 2012–March 2014), scoring availability of each food item on a scale from 0 to 4. Because data on diet and fecal samples were collected in subsequent years, we tested whether monthly phenology scores were correlated across study years using Spearman's rank correlation. Results indicated that patterns of food availability were similar in both years (Table S1).

### 2.4 Statistical analyses

Chao1 indices of alpha diversity (Chao, Chazdon, Colwell, & Shen, 2005) were calculated in QIIME v.1.9.1 (Caporaso et al., 2010). We used a linear mixed model (lmerTest package (Kuznetsova, Brockhoff, & Christensen, 2016), R v.3.2.4) to assess the impact of animal sex, age class, and sampling month on log‐transformed Chao1 richness, controlling for animal ID nested in group as a random factor. Nonsignificant interactions were excluded. The full model was compared to a null model containing only the random factor in a likelihood ratio test (R‐function “ANOVA,” method = “Chisq”). We also performed Spearman's rank correlations of mean monthly Chao1 indices with dietary measures, with false discovery rate (FDR) adjustment of *p*‐values (alpha‐level = 0.05). To determine whether female reproductive state influenced Chao1 richness, we performed Kruskal–Wallis tests for each sampling month, comparing Chao1 richness between adult nonreproducing females, adult males, and pregnant/lactating females.

To assess beta diversity, weighted (WUF) and unweighted Unifrac (UUF) distances (Lozupone, Hamady, Kelley, & Knight, 2007) were calculated in QIIME. WUF distances take both the phylogenetic relatedness and abundance of microbial taxa into account. We assessed differences in sample clustering patterns and microbial community composition according to group, sex, and age class using permutational analysis of variance (PERMANOVA, vegan package (Oksanen et al., 2016), R v.3.2.4) with 10,000 permutations based on WUF and UUF distances, stratifying by animal ID to control for repeated sampling. Because in the initial model, sampling month as well as social group and their interaction were significant predictors, we additionally ran a separate model for each month to assess whether the group effect was consistent throughout the study period. Interaction terms were excluded if not significant. Clustering patterns were visualized using principal coordinates analysis based on WUF distances.

Monthly differences in the abundance of bacterial phyla and families present in at least 10% of samples were assessed using Friedman tests, as data violated assumptions for parametric tests. Post hoc comparisons were carried out using Nemenyi tests (PMCMR package (Pohlert, 2014), R v.3.2.4). We used a series of Spearman's rank correlations to correlate mean monthly phylum and family abundances with mean monthly intake of fruits, mature leaves, young leaves, and flowers (in time spent feeding on these items relative to total feeding time), as well as mean monthly proportion of nonstructural carbohydrates, crude protein, fat and fiber in the diet, mean monthly total energy intake (kcal per hour of observation), and mean monthly dietary diversity (number of plant species fed on per observation day). *p*‐values were FDR‐adjusted. The same analyses were repeated for abundance of known genera using the genus‐level data subset.

## 3 RESULTS

**Figure 2:**
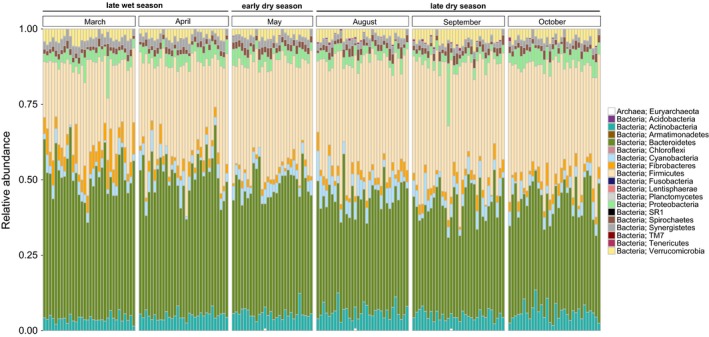
Relative abundance of microbial phyla in fecal samples (*N* = 187) of wild Verreaux's sifakas during the late wet, early dry, and late dry season

The rarefied data contained 2,617 unique OTUs (19 phyla, 160 families), while the number of unique OTUs per sample was on average 343.6 (*SD*: 34.66). A total of 654 unique OTUs were detected in all six sampling months, accounting for 99.2%–99.5% of the total monthly sequences. The dominating phyla throughout the sampling period were Bacteroidetes and Firmicutes, together accounting for 70%–80% of the microbiota (Figure 2).

**Figure 3:**
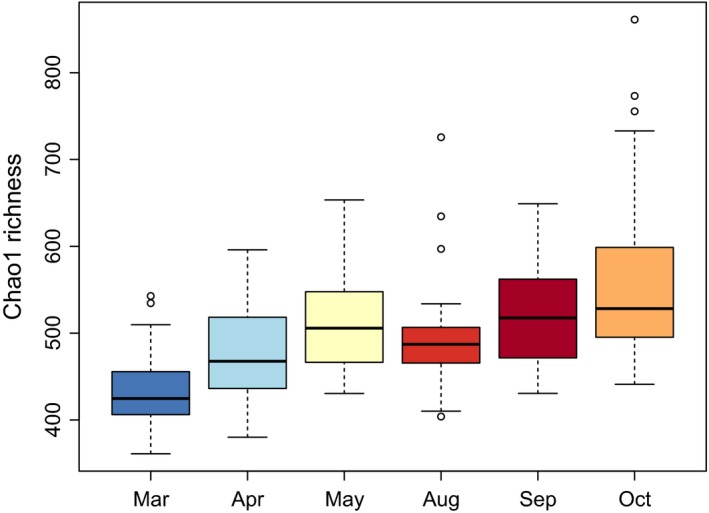
Boxplots presenting monthly differences in alpha diversity (Chao1 richness) of the fecal microbiota of wild Verreaux's sifakas

Chao1 richness estimates were similar between sifaka groups (Fig. S4), but showed significant monthly differences. Microbial diversity was lowest in March, when the animals consumed mostly fruit, increased toward the dry season, and was highest in October, when their diet was dominated by leaves (Table 1, Figure 3). In contrast, we did not find any significant effects of sex or age class on Chao1 estimates. Furthermore, lactating or pregnant females did not differ significantly in Chao1 richness from nonreproducing females or adult males in any month (Table S2). Mean monthly Chao1 richness was significantly correlated with the proportion of nonstructural carbohydrates in the diet (*n* = 6, Spearman's ρ = 1, FDR‐adjusted *q*‐value = 0.031, Table S3), but not with dietary diversity in terms of plant species or any of the other diet descriptors.

**Table 1:** Linear mixed model testing the effect of animal sex, age class (adult/juvenile), and sampling month on log‐transformed Chao1 estimates. Significant *p*‐values (<.05) are printed in bold. The full model was compared to a null model containing only the random factor in a likelihood ratio test: χ^2^ = 65.3, *df* = 7, *p* < .001

Factor	Estimate	Std. Error	*df*	*t*‐value	*p*‐value
Intercept	6.07	0.03	121.9	242.44	**<.001**
Sex (ref. male)	−0.01	0.02	29.4	0.6	.553
Age class (ref. juvenile)	−0.01	0.03	29.8	−0.45	.653
Month (April)	0.09	0.03	151.5	2.94	**.004**
Month (May)	0.17	0.03	153.4	5.37	**<.001**
Month (Aug)	0.12	0.03	150.8	4.16	**<.001**
Month (Sep)	0.17	0.03	150.8	5.86	**<.001**
Month (Oct)	0.24	0.03	150.8	8.13	**<.001**

To assess beta‐diversity, weighted (WUF) and unweighted Unifrac (UUF) distances were calculated. Within each season, differences in microbiota composition between samples collected longitudinally from the same individuals were smaller than differences between samples collected from different individuals (within‐individual mean UUF/WUF distance: 0.33/0.16 [wet season, *N* = 87], 0.35/0.15 [dry season, *N* = 96] vs. between‐individual mean UUF/WUF distance: 0.38/0.2 [wet, *N* = 4,009], 0.4/0.19 [dry, *N* = 4,464]). Mean within‐individual UUF distances were significantly larger in the wet season than in the dry season, whereas WUF distances were not (Wilcoxon test, *N* = 32, *V* = 375, *p* = .012 and *V* = 226, *p* = .68, respectively).

Microbial community composition was significantly influenced by month, group membership, age class, and sex (Table 2). Interestingly, the effect size for month was larger than the effect size for group in the model based on WUF distances ($R_{\mathrm{month}}^{2}$ = 0.21 vs. $R_{\mathrm{group}}^{2}$ = 0.15), whereas the opposite pattern was found in the model based on UUF distances ($R_{\mathrm{month}}^{2}$ = 0.06 vs. $R_{\mathrm{group}}^{2}$ = 0.11). This result indicates that differences between months are mainly driven by microbial abundance, whereas differences between groups are mainly due to the presence or absence of taxa. Indeed, only 513 OTUs were common to all groups (as compared to 654 which were common to all sampling months), which accounted for 97.6%–98.9% of the microbiota in each group. 83–169 OTUs were unique to each group (mean: 117.8). These were rare OTUs, comprising only 0.00008% of the total sequences on average. Compared to month and group membership, effect sizes of age class and sex were very small and comparable between WUF and UUF models ($R_{\mathrm{age}\;\mathrm{class}}^{2}$ = 0.005, $R_{\mathrm{sex}}^{2}$ = 0.008/0.009).

**Table 2:** PERMANOVAs testing the effect of sampling month, group membership, sex, and age class (adult/juvenile) on weighted and unweighted Unifrac distances. Significant *p*‐values (<.05) are printed in bold

Model	Term	*df*	SS	MS	F	*R* ^²^	*p*‐value
WUF distances	Month	5	0.926	0.185	15.299	0.217	**<.001**
	Group	7	0.655	0.094	7.731	0.154	**<.001**
	Age class	1	0.023	0.023	1.901	0.005	**<.001**
	Sex	1	0.031	0.031	2.598	0.007	**<.001**
	Sex:Age class	1	0.045	0.045	3.732	0.011	**<.001**
	Month:Group	35	0.620	0.018	1.465	0.146	**<.001**
	Group:Age class	4	0.113	0.037	3.109	0.026	**<.001**
	Group:Sex	7	0.323	0.046	3.815	0.076	**<.001**
	Residuals	126	1.525	0.012		0.358	
	Total	186	4.260			1.000	
UUF distances	Month	5	0.853	0.171	2.742	0.060	**<.001**
	Group	7	1.621	0.232	3.723	0.114	**<.001**
	Age class	1	0.071	0.071	1.142	0.005	**<.001**
	Sex	1	0.127	0.127	2.043	0.009	**<.001**
	Sex:Age class	1	0.140	0.140	2.258	0.01	**<.001**
	Month:Group	35	2.223	0.064	1.021	0.156	**<.001**
	Group:Age class	4	0.399	0.133	2.137	0.028	**<.001**
	Group:Sex	7	0.944	0.135	2.167	0.066	**<.001**
	Residuals	126	7.836	0.062		0.551	
	Total	186	14.213			1.000	

**Figure 4:**
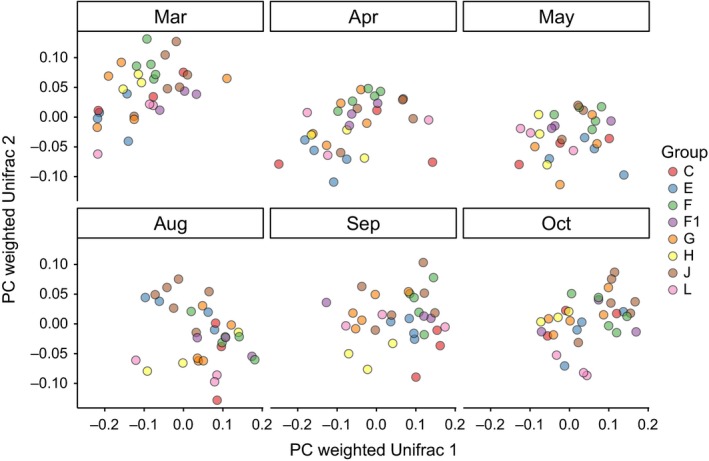
Principle coordinate analysis of the fecal microbiota of eight neighboring groups of Verreaux's sifakas during six different sampling months. The plot was generated using OTU‐level weighted Unifrac distances. Each dot represents one sample. Colors indicate group identity. The effect of group was significant in each month according to PERMANOVA analysis

In each month, group membership accounted for 35%–44% of the variance (monthly PERMANOVAs based on WUF distances, Table 3, Figure 4), whereas there were no significant effects of sex and age class in the monthly datasets. In addition, reproductive state did not influence microbiota composition among adult females (monthly PERMANOVAs based on WUF distances, controlling for group membership, data not shown). The results were similar when tests were performed based on UUF distances, but resulting in lower effect sizes (Table S4). Effect size of group membership was largest in August and smallest in March.

**Table 3:** PERMANOVAs for each monthly dataset testing the effect of group membership, sex, and age class (adult/juvenile) on weighted Unifrac distances. Significant *p*‐values (<.05) are printed in bold

Month	Term	*df*	SS	MS	F	*R* ^²^	*p*‐value
March	Group	7	0.217	0.031	1.729	0.347	**.013**
	Sex	1	0.011	0.011	0.596	0.017	.756
	Age class	1	0.004	0.004	0.226	0.006	.992
	Residuals	22	0.394	0.018		0.630	
	Total	31	0.626			1.000	
April	Group	7	0.210	0.030	1.725	0.350	**.028**
	Sex	1	0.018	0.018	1.034	0.030	.348
	Age class	1	0.006	0.006	0.368	0.011	.926
	Residuals	21	0.366	0.017		0.609	
	Total	30	0.601			1.000	
May	Group	7	0.152	0.022	1.857	0.393	**.007**
	Sex	1	0.018	0.018	1.510	0.046	.151
	Age class	1	0.007	0.007	0.608	0.018	.756
	Residuals	18	0.210	0.012		0.543	
	Total	27	0.387			1.000	
August	Group	7	0.245	0.035	2.583	0.440	**<.001**
	Sex	1	0.008	0.008	0.622	0.015	.724
	Age class	1	0.005	0.005	0.364	0.009	.947
	Residuals	22	0.298	0.014		0.536	
	Total	31	0.557			1.000	
September	Group	7	0.244	0.035	2.446	0.420	**<.001**
	Sex	1	0.005	0.004	0.316	0.008	.978
	Age class	1	0.019	0.019	1.338	0.033	.221
	Residuals	22	0.313	0.014		0.540	
	Total	31	0.580			1.000	
October	Group	7	0.205	0.029	1.811	0.352	**.005**
	Sex	1	0.011	0.011	0.686	0.019	.703
	Age class	1	0.011	0.011	0.696	0.019	.681
	Residuals	22	0.356	0.016		0.610	
	Total	31	0.584			1.000	

**Figure 5:**
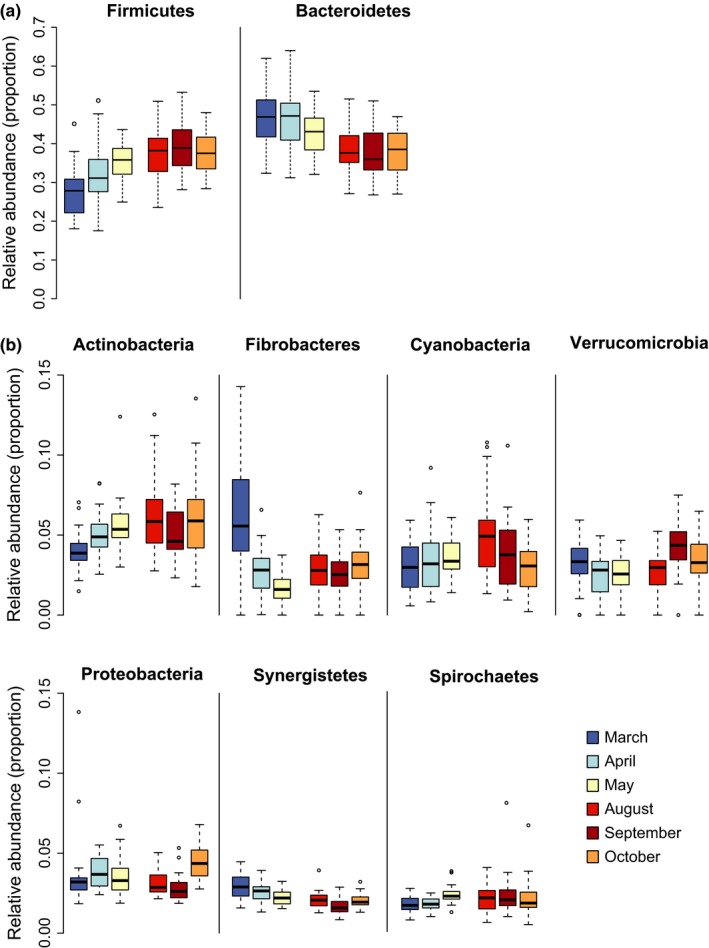
Boxplots presenting the relative abundance of (a) Firmicutes and Bacteroidetes and (b) less abundant phyla during March (*N* = 32), April (*N* = 31), May (*N* = 28), August (*N* = 32), September (*N* = 32), October (*N* = 32)

We detected statistically significant differences in monthly abundance in all 10 microbial phyla present in at least 10% of samples (Table 4). On the family level, 31 of 36 families showed significant differences in monthly abundance (Table S5). Specifically, dry season months (May, August–October) were characterized by higher abundances of Firmicutes (esp. unclassified Clostridiales, Lachnospiraceae, Ruminococcaceae) and Actinobacteria (esp. Coriobacteriaceae) as compared to March and April; and lower abundances of Bacteroidetes (esp. Bacteroidaceae, Paraprevotellaceae, unclassified Bacteroidales) and Synergistetes (Figure 5).

**Table 4:** Friedman tests and Nemenyi multiple comparisons testing the difference in monthly abundance of 10 bacterial phyla in the gut microbiota of wild Verreaux's sifakas. Significant *p*‐values (<.05) are printed in bold

Phylum	Friedman test			Nemenyi multiple comparison *p*‐values						Mean monthly relative abundance (%)
	χ^2^	*df*	*p*		March	April	May	August	September	
Actinobacteria	32.37	5	**<.001**	March	–	–	–	–	–	4.01
				April	.060	–	–	–	–	5.04
				May	**<.001**	.710	–	–	–	5.67
				August	**<.001**	.146	.918	–	–	6.26
				September	**.006**	.980	.980	.522	–	5.05
				October	**.001**	.830	1.000	.830	.996	6.02
Bacteroidetes	54.02	5	**<.001**	March	–	–	–	–	–	47.50
				April	.998	–	–	–	–	46.33
				May	.302	.570	–	–	–	42.78
				August	**<.001**	**<.001**	.087	–	–	38.42
				September	**<.001**	**<.001**	**.040**	1.000	–	37.64
				October	**<.001**	**<.001**	**.049**	1.000	1.000	37.97
Cyanobacteria	23.12	5	**<.001**	March	–	–	–	–	–	3.02
				April	.939	–	–	–	–	3.39
				May	.570	.980	–	–	–	3.55
				August	**.001**	**.021**	.146	–	–	4.99
				September	.618	.988	1.000	.124	–	3.90
				October	1.000	.918	.522	.000	.570	2.98
Fibrobacteres	54.56	5	**<.001**	March	–	–	–	–	–	6.30
				April	**.001**	–	–	–	–	2.68
				May	**.001**	**.040**	–	–	–	1.68
				August	**.001**	1.000	.060	–	–	2.82
				September	**.001**	1.000	**.040**	1.000	–	2.64
				October	**.002**	.996	**.008**	.988	.996	3.14
Firmicutes	51.39	5	**<.001**	March	–	–	–	–	–	27.46
				April	.302	–	–	–	–	31.81
				May	**.001**	.172	–	–	–	35.47
				August	**.001**	**.016**	.956	–	–	37.13
				September	**.001**	**.004**	.793	.998	–	38.97
				October	**.001**	**.021**	.970	1.000	.996	37.91
Proteobacteria	49.86	5	**<.001**	March	–	–	–	–	–	3.60
				April	.522	–	–	–	–	3.80
				May	1.000	.664	–	–	–	3.58
				August	.793	**.032**	.664	–	–	3.05
				September	.087	**<.001**	**.049**	.753	–	3.30
				October	**.003**	.342	**.006**	**<.001**	**<.001**	4.44
Spirochaetes	22.03	5	**<.001**	March	–	–	–	–	–	1.87
				April	1.000	–	–	–	–	1.85
				May	**.001**	**.001**	–	–	–	2.42
				August	.664	.641	.146	–	–	2.23
				September	.302	.283	.429	.993	–	2.35
				October	.879	.864	.054	.999	.929	2.10
Synergistetes	65.61	5	**<.001**	March	–	–	–	–	–	2.94
				April	.570	–	–	–	–	2.59
				May	.054	.847	–	–	–	2.26
				August	**<.001**	**.012**	.265	–	–	2.08
				September	**<.001**	**<.001**	**<.001**	.322	–	1.66
				October	**<.001**	**.010**	.248	1.000	.342	2.02
Tenericutes	40.14	5	**<.001**	March	–	–	–	–	–	0.06
				April	1.000	–	–	–	–	0.05
				May	.830	.753	–	–	–	0.11
				August	**.007**	**.004**	.215	–	–	0.28
				September	**<.001**	**<.001**	**.013**	.906	–	0.32
				October	.964	.929	.999	.087	**.003**	0.15
Verrucomicrobia	33.52	5	**<.001**	March	–	–	–	–	–	3.24
				April	.342	–	–	–	–	2.47
				May	.185	1.000	–	–	–	2.46
				August	.753	.988	.929	–	–	2.71
				September	.095	**<.001**	**<.001**	**.001**	–	4.14
				October	.993	.710	.498	.970	**.019**	3.28

On the phylum level, mean monthly relative abundances of Firmicutes were significantly negatively correlated with the proportion of feeding time dedicated to fruit (*n* = 6, *S* = 70, Spearman's ρ = −1, *q*‐value = 0.009), whereas we found significant positive correlations with fruit consumption for Bacteroidetes and Synergistetes (in both cases *n* = 6, *S* = 0, Spearman's ρ = 1, *q*‐value = 0.009). On the family level, only mean monthly abundance of Lachnospiraceae and Sphaerochaetaceae was significantly correlated with fiber intake (*n* = 6, *S* = 0, Spearman's ρ = 1, *q*‐value = 0.05 in both cases). There were no significant correlations between mean relative taxon abundances and the proportion of time spent feeding on mature leaves, young leaves, and flowers or intake of crude protein and nonstructural carbohydrates, at both phylum and family levels (Tables S6 and S7). Likewise, correlations with energy intake were not statistically significant.

In the data subset mapping to known sequences at a 94% threshold, 177 genera were identified. Significant differences in monthly abundance were detected in 22 of these (Table S8). For example, *Prevotella* and *Desulfovibrio* were more abundant during the wet season, while *Coprobacillus* was more abundant during the dry season. However, there were no statistically significant correlations with diet on the genus level (Table S9).

## 4 DISCUSSION

We demonstrated a clear pattern of seasonal variation in the intestinal microbiota of wild Verreaux's sifakas by sampling known individuals longitudinally during two distinct ecological seasons characterized by pronounced differences in rainfall, temperature, phenology, and sifaka diet. These seasonal patterns have already been reported in earlier studies on the same population (e.g., Norscia et al., 2006), indicating that they are generally stable across years. In line with dietary changes, we detected significant seasonal differences in microbial diversity as well as in the relative abundances of most microbial phyla and families present in the feces of sifakas. These changes in the relative abundance of taxa, especially concerning the Firmicutes‐Bacteroidetes ratio, could be linked to fruit and fiber consumption on the population level. Additionally, a significant effect of group membership was detected independent of sampling month. Age class and sex showed low effect sizes and, contrary to our prediction, no effect of reproductive state on microbiota composition was found.

### 4.1 Microbiota composition may be influenced by fruit and fiber intake

During the wet season, when sifakas consumed mostly fruit, the relative abundance of Firmicutes decreased while Bacteroidetes and Synergistetes increased in comparison with the dry season. Relative abundance of Firmicutes was negatively correlated with fruit intake while relative abundance of Bacteroidetes and Synergistetes was positively correlated with fruit intake. At the genus level, *Prevotella* increased markedly during months characterized by fruit consumption, similar to what has been found in western lowland gorillas (Gomez et al. 2016). In ruminants, *Prevotella* digest noncellulosic polysaccharides and pectin (White, Lamed, Bayer, & Flint, 2014), and in humans, high levels of *Prevotella* have been associated with a carbohydrate‐ and sugar‐rich diet (Wu et al., 2011).

The increase in Firmicutes in the dry season was mainly mediated by Lachnospiraceae, Ruminococcaceae, and other, unclassified, Clostridiales. In western lowland gorillas, Clostridiales also increased during times of low fruit intake (Gomez et al. 2016). Members of the Ruminococcaceae and Lachnospiraceae are efficient fermenters of fiber, such as cellulose or xylan, producing short‐chain fatty acids (SCFAs) in the process (Flint et al. 2012, Lynd, Weimer, van Zyl, & Pretorius, 2002). Lachnospiraceae were significantly positively correlated to fiber intake in our dataset, as were Sphaerochaetaceae, a family from the phylum Spirochaetes, which contains genera highly enriched in fermentation and carbohydrate metabolism genes. Interestingly, it was proposed that these genes have been acquired by gene transfer from the Clostridiales (Caro‐Quintero et al., 2012).

Whereas in humans, SCFAs provide around 6%–10% of the daily energy supply (Stevens & Hume, 1998), in folivorous primates this proportion can be as high as 57% (Popovich et al., 1997). Thus, microbial fermentation might compensate for reduced energy intake during the dry season, similar to what has been observed in black howler monkeys, which display an increase of Ruminococcaceae during reduced energy intake (Amato et al., 2015). Additionally, SCFAs reduce intestinal pH, making conditions even more unfavorable for Bacteroidetes (Duncan, Louis, Thomson, & Flint, 2009). Microbial fermentation in sifakas mainly takes place in the caecum and colon (Campbell, Eisemann, Williams, & Glenn, 2000; Campbell et al., 1999) and fecal microbiota are likely to resemble the microbiota in these distal gut compartments. Nevertheless, in future studies, microbiota analyses should be combined with measurement of fecal SCFA content in order to rigorously test these hypotheses.

A higher Firmicutes‐Bacteroidetes ratio, as observed here during the dry, lean season, has been shown to increase energy harvest from the diet in animal models (Turnbaugh et al., 2006, 2008), although recent studies have not supported the proposed link between the Firmicutes‐Bacteroidetes ratio and obesity in humans (Sze & Schloss, 2016). Nevertheless, in brown bears (*Ursus arctos*), an increase in Firmicutes and Actinobacteria and a decrease in Bacteriodetes were observed in summer, when the animals have to build up fat reserves (Sommer et al., 2016), supporting the potential functional link of this microbiota profile to increased energy extraction from the diet in wild animals. However, we did not observe a correlation of microbial taxa with our monthly estimate of energy intake, suggesting that, unlike in howler monkeys, compensation for lower energy intake may not be the main function of the seasonal changes. Instead, sifakas increase their intake of macronutrients during the dry season (Koch et al., 2017).

An alternative explanation for the increase in fermentative capacity during the dry season might be that female sifakas lactate and thus require more energy during this time. In female black howler monkeys, a higher Firmicutes‐Bacteroidetes ratio was found relative to males, potentially compensating for reproductive effort (Amato et al., 2014). As we did not observe any sex differences in microbiota composition during these months, this seems an unlikely explanation, indicating that seasonality impacts the gut microbiota independent of reproductive effort. Rather, females seem to compensate for their increased energy demand by increasing nutrient intake relative to males, at least during late lactation, the most energy‐demanding phase of reproduction (Richard, Dewar, Schwartz, & Ratsirarson, 2000).

On the family level, we only found few significant correlations with intake of specific food items or macronutrients, and none on the genus level. This may indicate that a diversity of different bacteria drives the phylum‐level correlations, which cannot be narrowed down to single genera or families. Closely related species may compete over similar ecological niches. For example, *Prevotella* and *Bacteroides*, both members of Bacteroidetes, commonly show a negative association with each other in the human gut (Faust et al., 2012). The relative abundances of these competing taxa may fluctuate in response to fine‐scale diet composition, for example, with regard to the type of fruit consumed. Alternatively, the fact that feeding data were collected a year prior to fecal sampling remains a limitation of the analysis and may have masked family‐ and genus‐level correlations. Due to this time‐lag between collection of feeding data and fecal samples, it was not possible to conduct analyses on the individual level. However, inter‐annual individual differences in diet intake are likely to even out on the population level, which is why population means were used here to analyze the influence of diet on microbiota composition. Nevertheless, both diet and microbiota composition vary by month; therefore, future studies should investigate individual‐level differences in diet and microbiota composition within each month to test these preliminary conclusions.

Furthermore, plant secondary compounds, which were not analyzed here, might also influence gut microbiota composition. However, our general understanding of the variety and functions of secondary compounds, as well as their impact on nutrient intake, is still very poor. Despite the negative impact on digestibility commonly attributed to secondary compounds, condensed tannins, for example, may increase crude protein flow to the intestine at low concentrations (Barry & Manley, 1984; Mangan, 1988; Waghorn, Ulyatt, John, & Fisher, 1987). Secondary compounds are expected to occur in higher concentrations in leaves than in fruits; however, in a study on gorilla diet, some leaves did not contain tannins while commonly eaten fruits did (Rothman et al., 2006). Therefore, future studies should investigate the potential impact of different classes of secondary compounds on the gut microbiome to begin unraveling their interactions.

### 4.2 Lipid metabolism may drive seasonal differences in Coriobacteriaceae

We also observed an increase in the relative abundance of Actinobacteria during the dry season, which was almost exclusively due to Coriobacteriaceae. Tight associations have been shown between the abundance of Coriobacteriaceae and the hosts’ lipid metabolism. Coriobacteriaceae were positively associated with liver triglyceride levels and serum cholesterol levels and negatively associated with liver glucose and glycogen levels in laboratory rodents (Claus et al., 2011). Additionally, cholesterol excreted in bile had antibacterial effects on Coriobacteriaceae (Martínez et al., 2013). Thus, an increase in endogenous lipid metabolism and/or a decrease of fecal cholesterol excretion in sifakas during the dry season could cause the increase in Coriobacteriaceae. Further studies on the fecal metabolome of sifakas are needed, however, to test this hypothesis.

### 4.3 Microbial diversity is linked to the amount of nonstructural carbohydrates in the diet

We detected a remarkable diversity of OTUs in the intestinal microbiota of wild Verreaux's sifakas, 95% of which could not be assigned to known sequences at the common 97% similarity level. This high percentage of unknown sequences is unlikely to be an artifact of sequencing, as the experimental protocol used significantly reduces sequencing errors (Gohl et al., 2016). Sequence quality scores as well as read lengths indicated that the generated sequences were of high quality. Furthermore, this high proportion of undescribed bacterial species is in line with previous results on the microbiota of wild lemurs (Fogel, 2015), suggesting a high level of endemism not only in Malagasy vertebrates but also in their microbiota (see also Supplementary Discussion). Similarly, high proportions of undescribed bacterial taxa have also been detected in other gut microbiota studies, including in human hunter‐gatherer populations (Schnorr et al., 2014).

Within‐individual microbial diversity, that is, alpha diversity, increased during the dry season and was correlated to the proportion of nonstructural carbohydrates in the animals’ diet, in line with the hypothesis that microbial diversity is predominantly driven by the diversity of different polysaccharides available for degradation (Martens, Kelly, Tauzin, & Brumer, 2014). In addition, we expected microbial diversity to vary with reproductive state. In humans, within‐individual microbial diversity decreases during pregnancy (Koren et al., 2012). However, no differences were observed between lactating or pregnant females, nonreproducing females and males during the months included in the study. However, samples were not available for the entire year and, thus, we may have missed changes occurring during late pregnancy, around birth or during late lactation. For example, sex differences in diet were most pronounced from November to January (Koch et al., 2017), a period for which no fecal samples were available.

### 4.4 Sex and age class are minor predictors of microbiota variation

Our predictions of sex and age class differences were only partly supported. Neither microbial diversity nor monthly microbiota composition were significantly influenced by sex or age class, although these factors showed weak significant effects in the combined dataset over all months.

Dietary divergence, as well as other differences, for example, in social behavior and physiology, was expected to generate divergence in microbiota composition. However, studies on wild mammals investigating sex and age class differences in microbiota composition have generally reported low effect sizes (Amato et al., 2014; Bennett et al., 2016; Tung et al., 2015). Large sample sizes may be needed to detect these effects, if they exist, which may explain why we found statistically significant effects in the model combining all samples, but not in the monthly data subsets. In addition, sifakas lack sexual dimorphism (Kappeler, 1990) and lemurs generally exhibit smaller sex differences in androgen levels than other mammals (Drea, 2007; von Engelhard, Kappeler, & Heistermann, 2000), indicating that sex differences in physiology may be generally less pronounced.

### 4.5 Microbiota composition is influenced by group membership

Group membership significantly influenced microbiota composition independent of sampling month. This was mainly due to the absence or presence of OTUs and in a lesser extent to differences in microbial abundance. These group differences may be explained by several factors, including differences in group composition, age structure, and social behavior. For example, social relationships as significant predictors of microbiota similarity have been found in baboons (Tung et al., 2015) and chimpanzees (Moeller et al., 2016). However, dietary differences exist even between neighboring sifaka groups, probably due to divergent food availability in each home range (unpublished data). This may be a better explanation for the group differences here, as the largest effect sizes of group membership on microbiota composition were observed during the dry season when home ranges contract and overlap less (Norscia et al., 2006), whereas group composition was stable across seasons and intergroup encounter rates do not vary seasonally (Koch, Signer, Kappeler, & Fichtel, 2016). Furthermore, animals are often more closely related within than between groups, although previous studies on wild primates have found little evidence for genetic relatedness as a determinant of gut microbiota similarity (Degnan et al., 2012; Moeller & Ochman, 2013). A follow‐up study is currently being conducted to disentangle these factors in Verreaux's sifakas.

## 5 CONCLUSIONS

We detected a clear pattern of seasonal variation in the microbiota of wild Verreaux's sifakas, by sampling known individuals longitudinally during two distinct ecological seasons. This variation may be due to dietary shifts, as indicated by significant correlations between the abundance of microbial taxa and diet composition on the population level. However, further studies including fecal metabolome data and individual‐level diet composition are needed to substantiate this preliminary conclusion. Together with findings from other mammals, our results demonstrate the plastic nature of the gut microbiota. This plasticity may have played a pivotal role during primate adaptation to different diets. Microbiota divergence between groups suggests that additionally to macroecological patterns, either small‐scale variation in diet/habitat or host genetic and social factors shape commensal microbial communities. In contrast, effects of sex, age, and reproductive state were less pronounced than expected. Further studies with larger sample sizes and sampling distributed over the entire year may be needed to detect less pronounced effects and to determine the factors driving the observed differences between groups.

## AUTHOR CONTRIBUTIONS

AS, CF, KRA, JBC, FK, GA, DK, and PMK conceived the ideas and wrote the manuscript; AS conducted the field work; DK, GA, and AS analyzed the data. All authors gave final approval for publication.

## DATA ACCESSIBILITY

Sequences have been deposited in the European Bioinformatics Institute database under project no. PRJEB20740. All additional data have been deposited in the Harvard Dataverse Repository under https://doi.org/10.7910/dvn/1h4nv2.

## CONFLICT OF INTEREST

None declared.
