# Supplementary material for: Patterns of seasonality and group membership characterize the gut microbiota in a longitudinal study of wild Verreaux's sifakas (Propithecus verreauxi)
Source: Ecol Evol. 2017 Jun 15;7(15):5732–45. doi: 10.1002/ece3.3148 (PMC5551086; doi:10.1002/ece3.3148)
Supplement: Supplementary file 1 [file ECE3-7-5732-s001.pdf]

Seasonality and group membership shape the gut microbiota in a longitudinal study of wild Verreaux's sifakas (*Propithecus verreauxi*)

Andrea Springer, Claudia Fichtel, Gabriel A. Al-Ghalith, Flávia Koch, Katherine R. Amato, Jonathan B. Clayton, Dan Knights and Peter M. Kappeler

Supplementary Material

Supplementary Methods

Primary/Secondary PCR Amplification:

The V4 region of the 16S rRNA was amplified using a two-step PCR protocol. The primary amplification was done using an ABI7900 qPCR machine. The following recipe was used: 3 µl template DNA, 0.48 µl nuclease-free water, 1.2 µl 5x KAPA HiFi buffer (Kapa Biosystems, Woburn, MA), 0.18 µl 10 mM dNTPs (Kapa Biosystems, Woburn, MA), 0.3 µl DMSO (Fisher Scientific, Waltham, MA), 0.12 µl ROX (25 µM) (Life Technologies, Carlsbad, CA), 0.003 µl 1000x SYBR Green, 0.12 µl KAPA HiFi Polymerase (Kapa Biosystems, Woburn, MA), 0.3 µl forward primer (10 µM), 0.3 µl reverse primer (10 µM). Cycling conditions were: 95°C for 5 minutes, followed by 25 cycles of 98°C for 20 seconds, 55°C for 15 seconds, and 72°C for 1 minute. The primers for the primary amplification contained both 16S-specific primers (Meta_V4_515F and Meta_V4_806R), as well as adapter tails for adding indices and Illumina flow cell adapters in a secondary amplification; and the sequences are as follows (16S-specific sequences in bold):
Meta_V4_515F
(TCGTCGGCAGCGTCAGATGTGTATAAGAGACAG**GTGCCAGCMGCCGCGGTAA**) and
Meta_V4_806R
(GTCTCGTGGGCTCGGAGATGTGTATAAGAGACAG**GGACTACHVGGGTWTCTAAT**).

The amplicons from the primary PCR were diluted 1:100 in sterile, nuclease-free water, and a second PCR reaction was set up to add the Illumina flow cell adapters and indices. The secondary amplification was done on a fixed block BioRad Tetrad PCR machine using the following recipe: 5 µl template DNA, 1 µl nuclease-free water, 2 µl 5x KAPA HiFi buffer (Kapa Biosystems, Woburn, MA), 0.3 µl 10 mM dNTPs (Kapa Biosystems, Woburn, MA), 0.5 µl DMSO (Fisher Scientific, Waltham, MA) 0.2 µl KAPA HiFi Polymerase (Kapa Biosystems, Woburn, MA), 0.5 µl forward primer (10 µM), 0.5 µl reverse primer (10 µM). Cycling conditions were: 95°C for 5 minutes, followed by 10 cycles of 98°C for 20 seconds, 55°C for 15 seconds, 72°C for 1 minute, followed by a final extension at 72°C for 10 minutes. The following indexing primers were used (X indicates the positions of the 8 bp indices):
Forward: AATGATACGGCGACCACCGAGATCTACACXXXXXXXXTCGTCGGCAGCGTC and
Reverse: CAAGCAGAAGACGGCATACGAGATXXXXXXXXGTCTCGTGGGCTCGG

Normalization and Sequencing:

The samples were normalized using a SequalPrep capture-resin bead plate (Life Technologies, Carlsbad, CA) and pooled using equal volume. The final pools were quantified via PicoGreen dsDNA

assay (Life Technologies, Carlsbad, CA) and diluted to 2nM. 10 µl of the 2 nM pool was denatured with 10 µl of 0.2 N NaOH, diluted to 8 pM in Illumina's HT1 buffer, spiked with 15% phiX, heat denatured at 96°C for 2 minutes, and sequenced using a MiSeq 600 cycle v3 kit (Illumina, San Diego, CA).

Supplementary Methods Discussion

OTU picking procedure:

The samples collected for this study were sequenced by the University of Minnesota Genomic Core (UMGC). It is notable that a very small proportion of reads (~5%) in this study mapped to known reference genomes in GreenGenes (DeSantis et al. 2006) 13.8 at 97% identity. To rule out the possibility of sequence contamination or invalid bioinformatics pipeline settings, multiple validation steps were conducted. The same Illumina MiSeq sequencing protocol was run on samples from other non-human primates, including samples of captive populations from Como Zoo. Approximately 80% of reads from these zoo samples mapped to the same database at 97% identity or higher (unpublished data). Amplification and read distributions were consistently high with a median around 32,000 sequences per sample after quality control (QC) in both cases. Quality control procedures followed field best practices and are the same that produced the higher read mapping rates for non-Sifaka primate communities. Specifically, the following observations were made and processes performed:

The UMGC quality report indicated consistently high quality platform and amplification performance. The fraction of chimeric sequences reported through their checks was low, and chimeric sequences are less of an issue when using closed-reference approaches provided the database itself is chimera-checked. Adaptor trimming was performed using standard procedures: Trimmomatic (Bolger et al. 2014) was used with "Nextera" adapter removal at default settings, retaining both pairs when possible. Very few reads (< 1%) were discarded as a result. Next, reads were stitched with the widely-used FLASH (Magoč et al. 2011) tool, where the minimum overlap was set to be 200bp and the maximum set to 320bp, which are sensible for targeting the V4 region with the UMGC dual-index approach, where we ideally expect 292-bp regions after stitching with fully redundant forward and reverse reads used to quality-correct one another. Almost all reads were stitched, and almost all of these (95%) indeed stitched at 292bp in proper "innie" (concordant primer) orientation (Figure S1). Both of these are good indicators of quality, as contaminants and non-target DNA would be expected to produce either poor stitching or a wide distribution of stitched read lengths indicative of a random distribution of forward and reverse read overlap sizes.

a)

```
[FLASH] Read combination statistics:
[FLASH]     Total pairs:      34005
[FLASH]     Combined pairs:   32295
[FLASH]     Uncombined pairs: 1710
[FLASH]     Percent combined: 94.97%
[FLASH]
[FLASH] Writing histogram files.
[FLASH]
[FLASH] FLASH v1.2.11 complete!
[FLASH] 0.723 seconds elapsed
```

b)

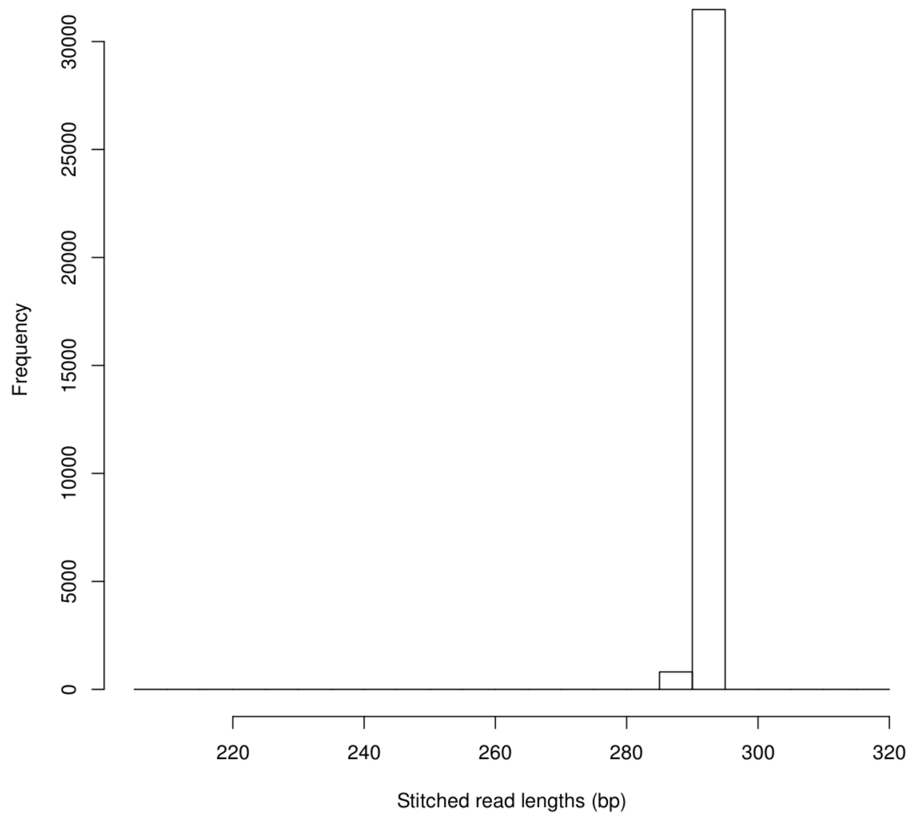

Figure S1: Distribution of stitched read lengths for a representative sample. a) Output from the FLASH program on the output from Trimmomatic shows approximately 95% of reads are stitched in the correct orientation ("outie" stitching is disabled). b) Mean combined read length was 291.6 and standard deviation was 0.879 (coefficient of variation = 0.003), showing high quality stitching at the expected read length. This is a positive quality indicator, as off-target sequences would be expected not to stitch, or to stitch at semi-random overlaps to produce a wide range of combined read lengths.

A final trimming pass on the stitched reads was performed to discard any residual contaminants near the end, but as expected in fully-contained reads, very few bases were discarded from either end when enforcing a quality minimum of 20 over 5 bases, and only 279 sequences were discarded for failing to maintain an average quality of 30 after trimming:

```
Trimming while quality < 20 on left, < 20 on right.
The average trimmed length was 290.59.
On average, the cut bases were: 0.84 (left), 0.16 (right).
The average read quality was 35.772.
```

De-duplication was also performed on these reads to measure whether certain amplicons appeared more than once, as would be expected of amplicon data. Within this sample alone, deduplication resulted in 19,767 unique sequences out of 32,015, which meets expectations that microbial communities have recurring members.

Furthermore, a subset of reads was randomly picked and checked with NCBI BLAST (Johnson et al. 2008). Figure S2a shows a representative BLAST result, demonstrating that this read mapped just under 97% identity to completely uncharacterized organisms. All of 50 spot-checked queries mapped full-length with no chimeric or partial reads, and all of them showed 99% or higher query coverage to at least one maximum-scoring hit. This result reveals that there is a dearth of information about read identity in these samples. Particularly notable is the fact that the same poorly-mapping sequence shown in Figure S2b (90% id) is found over 400 times in the dataset at high average quality (average PHRED33 > 35).

Alignments Download GenBank Graphics Distance tree of results

	Description	Max score	Total score	Query cover	E value	Ident	Accession
	Uncultured bacterium clone M125 16S ribosomal RNA gene, partial sequence	473	473	100%	8e-130	96%	HM124307.1
	Uncultured bacterium clone OK3_b04_1 16S ribosomal RNA gene, partial sequence	473	473	100%	8e-130	96%	EU468823.1
	Uncultured bacterium clone FF_f12_1 16S ribosomal RNA gene, partial sequence	451	451	100%	4e-123	95%	EU469726.1
	Uncultured bacterium clone FF_a03 16S ribosomal RNA gene, partial sequence	451	451	100%	4e-123	95%	EU469683.1
	Uncultured bacterium clone orang1_aai55d02 16S ribosomal RNA gene, partial sequence	435	435	100%	4e-118	94%	EU462476.1
	Uncultured bacterium clone orang1_aai55a10 16S ribosomal RNA gene, partial sequence	435	435	100%	4e-118	94%	EU462461.1
	Uncultured bacterium clone orang1_aai54d03 16S ribosomal RNA gene, partial sequence	435	435	100%	4e-118	94%	EU462427.1
	Uncultured bacterium clone COL_aai16g06 16S ribosomal RNA gene, partial sequence	435	435	100%	4e-118	94%	EU460215.1
	Uncultured bacterium clone COL_aai16a07 16S ribosomal RNA gene, partial sequence	435	435	100%	4e-118	94%	EU460180.1
	Uncultured bacterium clone COL_aai15f12 16S ribosomal RNA gene, partial sequence	435	435	100%	4e-118	94%	EU460165.1
	Uncultured bacterium clone COL_aai15e07 16S ribosomal RNA gene, partial sequence	435	435	100%	4e-118	94%	EU460153.1
	Uncultured bacterium clone COL_aai15b12 16S ribosomal RNA gene, partial sequence	435	435	100%	4e-118	94%	EU460133.1
	Uncultured bacterium clone COL_aai13f09 16S ribosomal RNA gene, partial sequence	435	435	100%	4e-118	94%	EU460053.1
	Uncultured bacterium clone COL_aai13c08 16S ribosomal RNA gene, partial sequence	435	435	100%	4e-118	94%	EU460035.1
	Uncultured bacterium clone COL_aai13b12 16S ribosomal RNA gene, partial sequence	431	431	99%	5e-117	93%	EU460030.1
	Uncultured bacterium clone COL_aai16c09 16S ribosomal RNA gene, partial sequence	429	429	100%	2e-116	93%	EU460193.1
	Uncultured bacterium clone COL_aai16a02 16S ribosomal RNA gene, partial sequence	429	429	100%	2e-116	93%	EU460176.1
	Uncultured bacterium clone COL_aai13a04 16S ribosomal RNA gene, partial sequence	429	429	100%	2e-116	93%	EU460020.1
	Uncultured bacterium clone COL_aai16b01 16S ribosomal RNA gene, partial sequence	424	424	100%	8e-115	93%	EU460186.1
	Uncultured bacterium clone LNH_9_9_11_Water.204989 16S ribosomal RNA gene, partial sequence	407	407	100%	8e-110	92%	KM133274.1
	Uncultured bacterium clone LNH_9_9_11_Water.193009 16S ribosomal RNA gene, partial sequence	407	407	100%	8e-110	92%	KM132917.1
	Uncultured bacterium clone LNH_9_9_11_Water.3998 16S ribosomal RNA gene, partial sequence	407	407	100%	8e-110	92%	KM127035.1
	Uncultured bacterium clone 8LP_003b_c10F03 16S ribosomal RNA gene, partial sequence	407	407	100%	8e-110	92%	JX671394.1
	Uncultured bacterium clone F4079 16S ribosomal RNA gene, partial sequence	401	401	100%	4e-108	91%	KX504648.1

a)

	Description	Max score	Total score	Query cover	E value	Ident	Accession
	Uncultured bacterium clone WA_aaa03g07 16S ribosomal RNA gene, partial sequence	374	374	100%	8e-100	90%	EU473506.1
	Uncultured bacterium clone WA_aaa04e09 16S ribosomal RNA gene, partial sequence	374	374	100%	8e-100	90%	EU473448.1
	Uncultured bacterium gene for 16S rRNA, partial sequence, clone: OSTca204	374	374	100%	8e-100	90%	AB386077.1
	Uncultured Porphyromonadaceae bacterium clone 3483 16S ribosomal RNA gene, partial sequence	370	370	99%	1e-98	90%	KP105719.1
	Uncultured Parabacteroides sp. clone 2614 16S ribosomal RNA gene, partial sequence	370	370	99%	1e-98	90%	KP105099.1
	Uncultured Porphyromonadaceae bacterium clone 2227 16S ribosomal RNA gene, partial sequence	370	370	99%	1e-98	90%	KP104783.1
	Uncultured bacterium gene for 16S rRNA, partial sequence, clone: OSTca227	368	368	100%	4e-98	89%	AB386097.1
	Uncultured bacterium gene for 16S rRNA, partial sequence, clone: OSTca083	368	368	100%	4e-98	89%	AB385960.1
	Uncultured bacterium gene for 16S rRNA, partial sequence, clone: OSTca077	368	368	100%	4e-98	89%	AB385955.1
	Uncultured bacterium gene for 16S rRNA, partial sequence, clone: OSTca066	368	368	100%	4e-98	89%	AB385944.1
	Uncultured bacterium clone M01598_122_000000000-ADV8A_1_2113_8126_4784 16S ribosomal RNA gene, partial sequenc	366	366	99%	1e-97	89%	KR936022.1
	Uncultured bacterium clone 186139-17 16S ribosomal RNA gene, partial sequence	366	366	100%	1e-97	90%	JN857627.1
	Uncultured Porphyromonadaceae bacterium clone 2607 16S ribosomal RNA gene, partial sequence	364	364	99%	5e-97	90%	KP105093.1
	Uncultured bacterium clone 955 16S ribosomal RNA gene, partial sequence	363	363	100%	2e-96	89%	KU506169.1

b)

Figure S2. Representative BLAST searches of random sequences drawn from the test sample. a) Not only are there no matches above 97% identity, but all matches as low as 91% identity hit uncultured, unclassified organisms without attendant taxonomic information. b) This read does not hit any records above 90% identity, yet 4 of the 4 annotated matches above 89% match family Porphyromonadaceae (including the result for species Parabacteroides, which is also in family Porphyromonadaceae), implying that it is possible to apply taxonomic labels reliably, but not with finer granularity than family level.

To mitigate this problem, there are a few available options: First, de novo or "hybrid"/"open-reference" clustering could be used. There is little benefit in using an open reference pipeline if 95% of reads will inevitably end up as de novo clusters, and de novo clusters are themselves reliant on database-based methods for taxonomic classification (Caporaso et al. 2010), as all classifiers must use existing knowledge or training data to assign taxonomic identity to new data. Further, because OTUs generated by de novo methods are not comparable across studies due to the stochastic nature of their selection, and because interpreting de novo OTUs taxonomically requires some form of classification or database matching anyway, it is sensible to align the reads at lower identity and interpolate their taxonomies as exemplified in Figure S3. Although this is time-consuming, high-fidelity pipelines such as NINJA-OPS (Al-Ghalith et al. 2016) have demonstrated the ability to produce trustworthy assignments of short amplicon reads, and the existence of various levels of GreenGenes representative cluster databases with taxonomic trees for each of these levels, opens the opportunity to analyze the reads using higher-level taxonomic features.

The biological rationale behind the high proportion of unknown sequences, which does not appear to be artifactual under known quality metrics, is debatable. One hypothesis is that because Verreaux's sifakas are foraging herbivores on the remote island of Madagascar, where there has been little microbial characterization to date, their gut may simply present a distinct selective environment for species that have not been included in the creation of previous databases. Specifically, recent estimates report that 92-100% of all terrestrial animals on the island are endemic, and describe the island as one of the world's most critical hotspots of biodiversity (Goodman et al. 2005). It would not be out of expectation that the precise characterization of microbial life therein therefore presents challenges at familiar levels of assignment specificity, but an approach that weds traditional alignments with taxonomic assignment for each read is appropriate to overcome these challenges (Liu et al. 2008) while maintaining the ability to compare OTUs and taxa across studies.

References

Al-Ghalith, G.A., Montassier, E., Ward, H.N. & Knights, D. (2016) NINJA-OPS: Fast accurate marker gene alignment using concatenated ribosomes. *PLoS Comput Biol*, **12,** e1004658.

Bolger, A. M., Lohse, M., & Usadel, B. (2014). Trimmomatic: a flexible trimmer for Illumina sequence data. Bioinformatics, btu170.

Caporaso, J.G., Kuczynski, J., Stombaugh, J., Bittinger, K., Bushman, F.D., Costello, E.K., Fierer, N., Pena, A.G., Goodrich, J.K., Gordon, J.I., Huttley, G.A., Kelley, S.T., Knights, D., Koenig, J.E., Ley, R.E., Lozupone, C.A., McDonald, D., Muegge, B.D., Pirrung, M., Reeder, J., Sevinsky, J.R., Turnbaugh, P.J., Walters, W.A., Widmann, J., Yatsunenko, T., Zaneveld, J. & Knight, R. (2010) QIIME allows analysis of high-throughput community sequencing data. *Nature Methods*, **7,** 335-336.

DeSantis, T.Z., Hugenholtz, P., Larsen, N., Rojas, M., Brodie, E.L., Keller, K., Huber, T., Dalevi, D., Hu, P. and Andersen, G.L. (2006). Greengenes, a chimera-checked 16S rRNA gene database and workbench compatible with ARB. Applied and environmental microbiology, 72, 5069-5072.

Goodman, S. M., & Benstead, J. P. (2005). Updated estimates of biotic diversity and endemism for Madagascar. Oryx, 39, 73-77.

Johnson, M., Zaretskaya, I., Raytselis, Y., Merezhuk, Y., McGinnis, S., & Madden, T. L. (2008). NCBI BLAST: a better web interface. Nucleic acids research, 36(suppl 2), W5-W9.

Liu, Z., DeSantis, T. Z., Andersen, G. L., & Knight, R. (2008). Accurate taxonomy assignments from 16S rRNA sequences produced by highly parallel pyrosequencers. Nucleic acids research, 36, e120-e120.

Magoč, T., & Salzberg, S. L. (2011). FLASH: fast length adjustment of short reads to improve genome assemblies. Bioinformatics, 27, 2957-2963.

Supplementary Results

Figure S3: Rarefaction plot showing the phylogenetic diversity at different read depths within each sample. The metric PD_whole_tree is Faith's Phylogenetic Diversity, which adds up all the branch lengths as a measure of diversity, and was calculated using the QIIME pipeline.

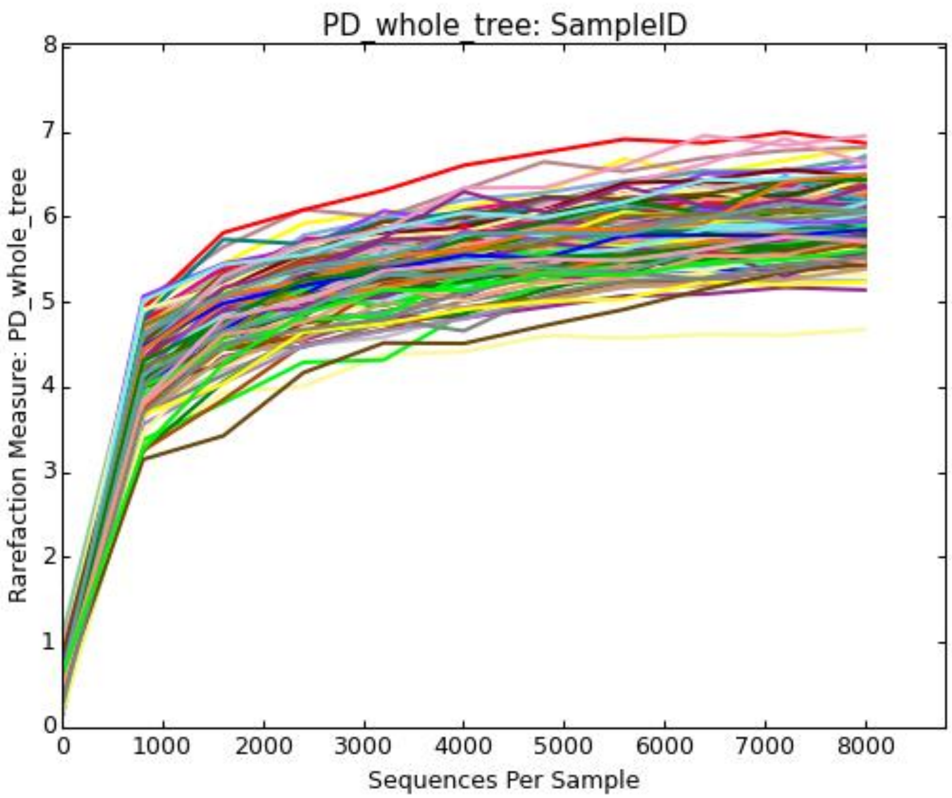

Figure S4: Chao1 richness estimates per sifaka group.

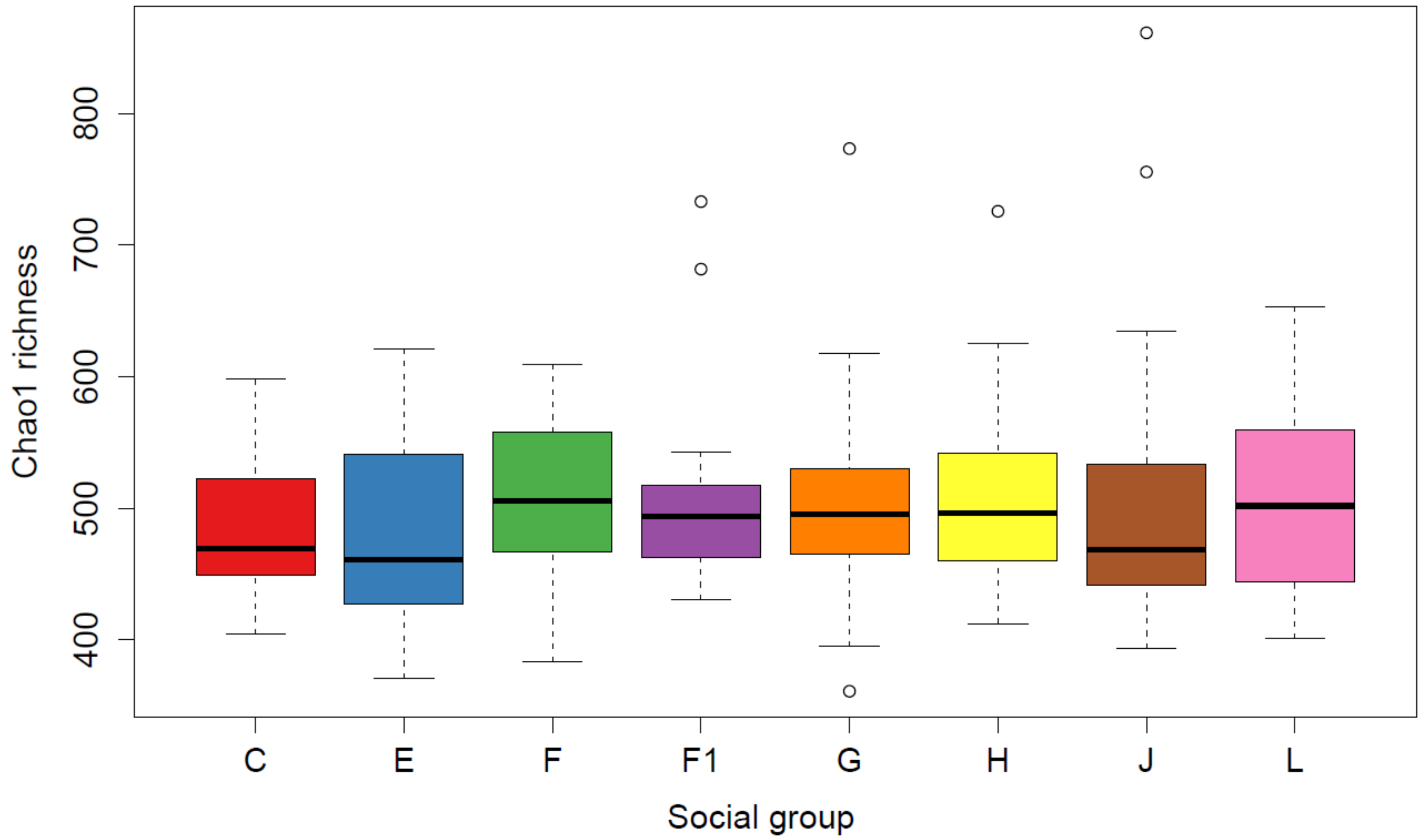

Table S1: Results of Spearman rank correlations of phenology scores for 692 trees across study years. Significant correlations are printed in bold (FDR-adjusted q ≤ 0.05).

	**March 2013 / March 2014**		**April 2012 / April 2014**		**May 2012 / May 2014**		**August 2012 / August 2013**		**Sept. 2012 / Sept. 2013**		**Oct. 2012 / Oct. 2013**	
	Spearman rho	q-value	Spearman rho	q-value	Spearman rho	q-value	Spearman rho	q-value	Spearman rho	q-value	Spearman rho	q-value
**young leaves**	-0.09	**0.014**	0.23	**<0.001**	0.5	**<0.001**	0.73	**<0.001**	0.68	**<0.001**	0.52	**<0.001**
**mature leaves**	0.15	**<0.001**	0.05	0.226	0.48	**<0.001**	0.74	**<0.001**	0.83	**<0.001**	0.71	**<0.001**
**fruits**	0.21	**<0.001**	0.05	0.226	0.23	**<0.001**	0.17	**<0.001**	0.29	**<0.001**	0.14	**<0.001**
**flowers**	0.43	**<0.001**	0.31	**<0.001**	0.15	**<0.001**	0.29	**<0.001**	0.12	**0.002**	0.16	**<0.001**

Table S2: Results of Kruskal-Wallis-tests assessing differences in Chao1 richness between males, adult non-reproducing females and adult reproducing (lactating/pregnant) females.

	**N**	**df**	**X^2**	**P-value**
**March**	27	2	4.06	0.13
**April**	26	2	0.37	0.83
**May**	24	2	0.91	0.64
**August**	27	2	1.29	0.52
**September**	27	2	0.16	0.92
**October**	27	2	1.56	0.46

Table S3: Results of Spearman rank correlations testing the association of mean monthly Chao1 richness with diet measures. Sample size was 6 in all cases. Significant correlations are printed in bold (FDR-adjusted q ≤ 0.05).

	**Spearman rho**	**q-value**
**Fruit intake** (min / hr of feeding time)	-0.89	0.122
**Intake of mature leaves** (min / hr of feeding time)	-0.03	1
**Intake of young leaves** (min / hr of feeding time)	0.37	0.724
**Intake of flowers** (min / hr of feeding time)	0.26	0.724
**Intake of TNCE*** (% of macronutrient intake)	**1**	**0.031**
**Intake of crude protein** (% of macronutrient intake)	0.43	0.724
**Intake of fiber** (% of macronutrient intake)	0.48	0.724
**Intake of fat** (% of macronutrient intake)	-0.94	0.092
**Overall energy intake** (kcal / hr)	-0.26	0.724
**Plant species consumed / month**	0.26	0.724

*TNCE = non-structural carbohydrates

Table S4: Results of PERMANOVAs testing the effect of group membership, sex and age class (adult or juvenile) on unweighted Unifrac distances. Significant P-values (< 0.05) are printed in bold.

Month	Term	Df	SS	MS	F	R^2	P
March	Group	7	0.633	0.090	1.417	0.293	**<0.001**
	Sex	1	0.068	0.068	1.059	0.031	0.314
	Age class	1	0.052	0.052	0.818	0.024	0.865
	Residuals	22	1.405	0.064		0.651	
	Total	31	2.158			1.000	
April	Group	7	0.626	0.089	1.498	0.309	**<0.001**
	Sex	1	0.079	0.079	1.319	0.039	**0.039**
	Age class	1	0.066	0.066	1.102	0.032	0.251
	Residuals	21	1.254	0.060		0.619	
	Total	30	2.025			1.000	
May	Group	7	0.597	0.085	1.282	0.313	**0.001**
	Sex	1	0.063	0.063	0.946	0.033	0.607
	Age class	1	0.053	0.053	0.789	0.028	0.923
	Residuals	18	1.198	0.067		0.627	
	Total	27	1.911			1.000	
August	Group	7	0.589	0.084	1.225	0.265	**0.001**
	Sex	1	0.073	0.073	1.063	0.033	0.310
	Age class	1	0.050	0.050	0.733	0.023	0.982
	Residuals	22	1.511	0.069		0.680	
	Total	31	2.224			1.000	
September	Group	7	0.645	0.092	1.357	0.284	**<0.001**
	Sex	1	0.076	0.076	1.116	0.033	0.192
	Age class	1	0.057	0.057	0.839	0.025	0.869
	Residuals	22	1.495	0.068		0.658	
	Total	31	2.273			1.000	
October	Group	7	0.751	0.107	1.253	0.271	**0.004**
	Sex	1	0.062	0.062	0.720	0.022	0.978
	Age class	1	0.073	0.073	0.847	0.026	0.812
	Residuals	22	1.884	0.086		0.680	
	Total	31	2.769			1.000	

Table S5: Results of family-level analyses of monthly abundance. Families for which significant differences in monthly abundance were found are printed in bold. Significant P-values (< 0.05) are printed in italics.

Family	Friedman test					Nemenyi pair-wise comparison P-values					mean monthly relative abundance	Boxplot of monthly relative abundance
	X^2	df	P	FDR adjusted P		March	April	May	August	September		Mar April May Aug Sept Oct
**Coriobacteriaceae**	32.37	5	*<0.001*	*<0.001*	**March**	-	-	-	-	-	3.999%	
					**April**	0.060	-	-	-	-	5.029%	
					**May**	*<0.001*	0.710	-	-	-	5.646%	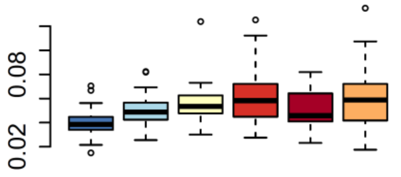
					**August**	*<0.001*	0.146	0.918	-	-	6.247%	
					**September**	*0.006*	0.980	0.980	0.522	-	5.039%	
					**October**	*0.001*	0.830	1.000	0.830	0.996	6.010%	
unclass. Actinobacteria	13.55	5	*0.019*	*0.02*	**March**	-	-	-	-	-	0.007%	
					**April**	0.850	-	-	-	-	0.003%	
					**May**	0.860	0.190	-	-	-	0.013%	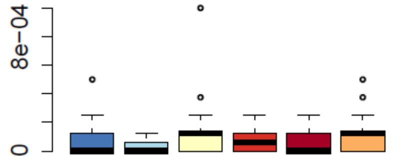
					**August**	0.960	0.340	1.000	-	-	0.008%	
					**September**	0.980	1.000	0.450	0.660	-	0.007%	
					**October**	0.930	0.270	1.000	1.000	0.570	0.010%	
**unclass. Bacteroidales**	32.41	5	*<0.001*	*<0.001*	**March**	-	-	-	-	-	15.050%	
					**April**	0.342	-	-	-	-	13.736%	
					**May**	0.893	0.939	-	-	-	13.749%	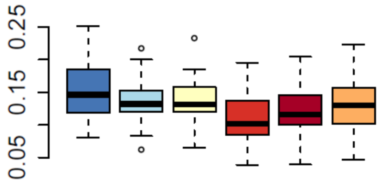
					**August**	*<0.001*	*0.040*	*0.002*	-	-	10.924%	
					**September**	*0.002*	0.429	0.060	0.893	-	11.855%	
					**October**	0.200	1.000	0.830	0.087	0.618	13.046%	
Bacteroidales f. BS11	6.89	5	0.23	0.23	**March**	-	-	-	-	-	0.001%	
					**April**	1.000	-	-	-	-	0.002%	
					**May**	0.930	0.960	-	-	-	0.004%	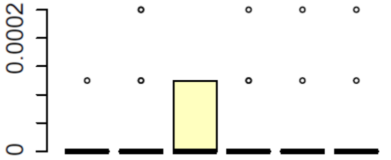
					**August**	1.000	1.000	0.770	-	-	0.002%	
					**September**	1.000	1.000	0.990	0.980	-	0.003%	
					**October**	1.000	1.000	0.960	1.000	1.000	0.002%	

Table S5 continued.

Family	Friedman test					Nemenyi pair-wise comparison P-values					mean monthly relative abundance	Boxplot of monthly relative abundance
	X^2	df	P	FDR adjusted P		March	April	May	August	September		Mar April May Aug Sept Oct
**Bacteroidaceae**	22.01	5	*<0.001*	*<0.001*	**March**	-	-	-	-	-	7.296%	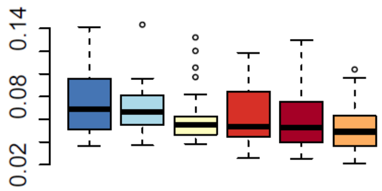
					**April**	1.000	-	-	-	-	6.980%	
					**May**	0.830	0.731	-	-	-	6.239%	
					**August**	0.879	0.793	1.000	-	-	6.385%	
					**September**	0.406	0.302	0.984	0.970	-	5.785%	
					**October**	*0.001*	*0.001*	0.080	0.060	0.342	5.299%	
**Porphyromonadaceae**	31.2	5	*<0.001*	*<0.001*	**March**	-	-	-	-	-	0.840%	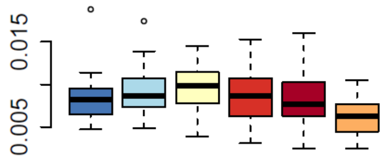
					**April**	0.830	-	-	-	-	0.925%	
					**May**	0.231	0.918	-	-	-	0.964%	
					**August**	0.999	0.618	0.104	-	-	0.855%	
					**September**	1.000	0.893	0.302	0.996	-	0.831%	
					**October**	*0.032*	*<0.001*	*<0.001*	0.087	*0.021*	0.634%	
**Prevotellaceae**	18.14	5	*0.003*	*0.003*	**March**	-	-	-	-	-	11.145%	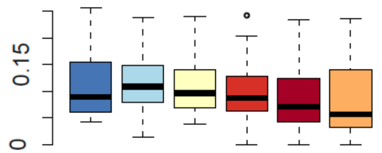
					**April**	0.996	-	-	-	-	11.556%	
					**May**	1.000	0.970	-	-	-	10.978%	
					**August**	1.000	0.970	1.000	-	-	10.020%	
					**September**	0.231	0.072	0.384	0.384	-	8.756%	
					**October**	*0.049*	*0.010*	0.104	0.104	0.988	8.673%	
**Bacteroidales f. RF16**	20.26	5	*0.001*	*0.001*	**March**	-	-	-	-	-	0.063%	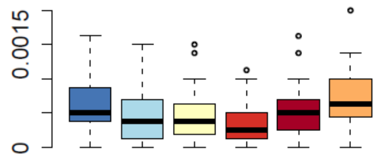
					**April**	0.710	-	-	-	-	0.047%	
					**May**	0.475	0.999	-	-	-	0.047%	
					**August**	0.104	0.864	0.970	-	-	0.036%	
					**September**	0.957	0.993	0.939	0.522	-	0.052%	
					**October**	0.793	0.072	*0.026*	*0.002*	0.265	0.071%	

Table S5 continued.

Family	Friedman test X^2	df	P	FDR adjusted P		Nemenyi pair-wise comparison P-values March	April	May	August	September	mean monthly relative abundance	Boxplot of monthly relative abundance (Mar, April, May, Aug, Sept, Oct)
**Bacteroidales f. S24-7**	36.33	5	*<0.001*	*<0.001*	**March**	-	-	-	-	-	1.871%	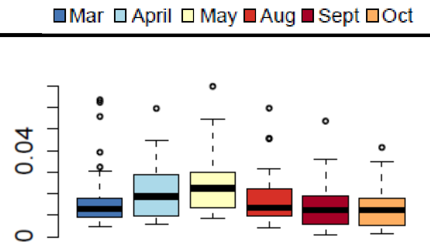
					**April**	0.664	-	-	-	-	2.118%	
					**May**	0.146	0.939	-	-	-	2.509%	
					**August**	0.956	0.172	*0.013*	-	-	1.721%	
					**September**	0.172	*0.002*	*<0.001*	0.664	-	1.414%	
					**October**	0.342	*0.006*	*<0.001*	0.863	0.999	1.395%	
**[Paraprevotellaceae]**	38.25	5	*<0.001*	*<0.001*	**March**	-	-	-	-	-	11.232%	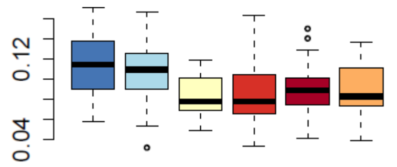
					**April**	1.000	-	-	-	-	10.963%	
					**May**	*0.005*	*0.002*	-	-	-	8.284%	
					**August**	*<0.001*	*<0.001*	0.970	-	-	8.471%	
					**September**	*0.013*	*0.006*	1.000	0.893	-	8.944%	
					**October**	*0.021*	*0.010*	0.998	0.830	1.000	8.848%	
**unclass. Cyanobacteria**	22.98	5	*<0.001*	*<0.001*	**March**	-	-	-	-	-	3.002%	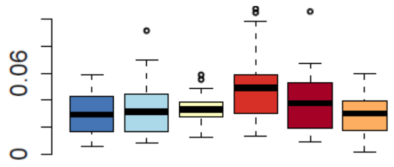
					**April**	0.939	-	-	-	-	3.342%	
					**May**	0.710	0.996	-	-	-	3.400%	
					**August**	*<0.001*	*0.016*	0.072	-	-	4.985%	
					**September**	0.618	0.988	1.000	0.104	-	3.891%	
					**October**	1.000	0.956	0.753	*0.001*	0.664	2.955%	
**unclass. Streptophyta**	75.08	5	*<0.001*	*<0.001*	**March**	-	-	-	-	-	0.017%	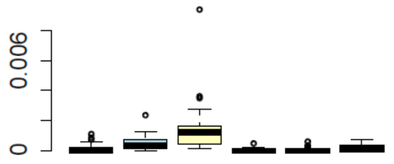
					**April**	*0.032*	-	-	-	-	0.049%	
					**May**	*<0.001*	0.215	-	-	-	0.153%	
					**August**	0.995	*0.005*	*<0.001*	-	-	0.008%	
					**September**	0.546	*<0.001*	*<0.001*	0.863	-	0.005%	
					**October**	0.906	0.363	*<0.001*	0.618	0.072	0.021%	

Table S5 continued.

**Family**	**Friedman test**				**Nemenyi pair-wise comparison P-values**						**mean monthly relative abundance**	**Boxplot of monthly relative abundance**
	**X^2**	**df**	**P**	**FDR adjusted P**		**March**	**April**	**May**	**August**	**September**		Mar April May Aug Sept Oct
**Fibrobacteraceae**	54.56	5	*<0.001*	*<0.001*	**March**	-	-	-	-	-	6.297%	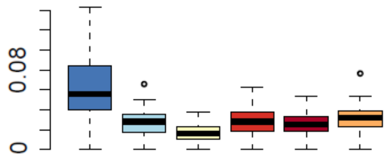
					**April**	*<0.001*	-	-	-	-	2.677%	
					**May**	*<0.001*	*0.040*	-	-	-	1.677%	
					**August**	*<0.001*	1.000	0.060	-	-	2.823%	
					**September**	*<0.001*	1.000	*0.040*	1.000	-	2.640%	
					**October**	*0.002*	0.996	*0.008*	0.988	0.996	3.138%	
Streptococcaceae	12.38	5	*0.03*	*0.03*	**March**	-	-	-	-	-	0.001%	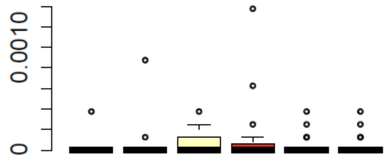
					**April**	0.960	-	-	-	-	0.004%	
					**May**	0.280	0.810	-	-	-	0.006%	
					**August**	0.960	1.000	0.810	-	-	0.009%	
					**September**	0.980	1.000	0.710	1.000	-	0.004%	
					**October**	0.990	1.000	0.690	1.000	1.000	0.003%	
**unclass. Clostridiales**	56.74	5	*<0.001*	*<0.001*	**March**	-	-	-	-	-	10.186%	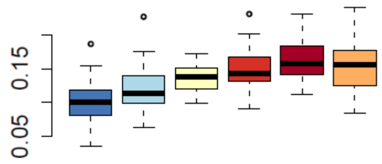
					**April**	0.429	-	-	-	-	11.885%	
					**May**	*0.003*	0.429	-	-	-	13.536%	
					**August**	*<0.001*	*0.013*	0.710	-	-	14.853%	
					**September**	*<0.001*	*<0.001*	0.104	0.863	-	16.363%	
					**October**	*<0.001*	*0.008*	0.618	1.000	0.918	15.629%	
**Clostridiaceae**	15.07	5	*0.01*	*0.01*	**March**	-	-	-	-	-	0.021%	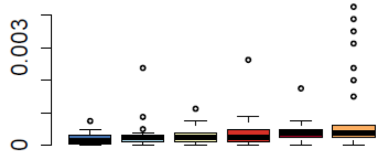
					**April**	0.984	-	-	-	-	0.028%	
					**May**	0.594	0.939	-	-	-	0.031%	
					**August**	0.342	0.773	0.999	-	-	0.038%	
					**September**	0.060	0.283	0.847	0.970	-	0.041%	
					**October**	*0.032*	0.185	0.731	0.918	1.000	0.089%	

Table S5 continued.

**Family**	**Friedman test**					**Nemenyi pair-wise comparison P-values**					**mean monthly relative abundance**	**Boxplot of monthly relative abundance**
	**X^2**	**df**	**P**	**FDR adjusted P**		**March**	**April**	**May**	**August**	**September**		Mar April May Aug Sept Oct
**Eubacteriaceae**	27.47	5	*<0.001*	*<0.001*	**March**	-	-	-	-	-	0.013%	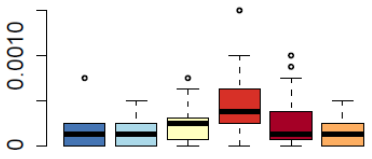
					**April**	0.996	-	-	-	-	0.016%	
					**May**	0.498	0.812	-	-	-	0.024%	
					**August**	*<0.001*	*0.002*	0.095	-	-	0.045%	
					**September**	0.570	0.863	1.000	0.072	-	0.024%	
					**October**	0.995	1.000	0.830	*0.002*	0.879	0.014%	
**Lachnospiraceae**	55.92	5	*<0.001*	*<0.001*	**March**	-	-	-	-	-	6.717%	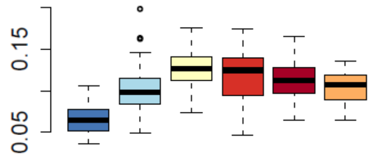
					**April**	*0.005*	-	-	-	-	10.142%	
					**May**	*<0.001*	*0.016*	-	-	-	12.569%	
					**August**	*<0.001*	0.302	0.863	-	-	11.627%	
					**September**	*<0.001*	0.546	0.641	0.999	-	11.380%	
					**October**	*0.001*	0.997	0.066	0.594	0.830	10.416%	
**Ruminococcaceae**	46.12	5	*<0.001*	*<0.001*	**March**	-	-	-	-	-	3.259%	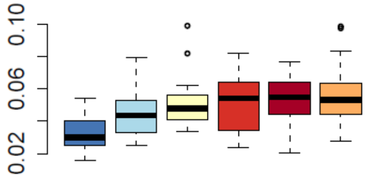
					**April**	*0.032*	-	-	-	-	4.391%	
					**May**	*<0.001*	0.522	-	-	-	5.096%	
					**August**	*<0.001*	*0.040*	0.830	-	-	5.163%	
					**September**	*<0.001*	0.342	1.000	0.939	-	5.377%	
					**October**	*<0.001*	0.384	1.000	0.918	1.000	5.566%	
**Veillonellaceae**	51.67	5	*<0.001*	*<0.001*	**March**	-	-	-	-	-	7.116%	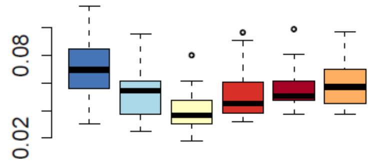
					**April**	*0.004*	-	-	-	-	5.181%	
					**May**	*<0.001*	0.080	-	-	-	3.895%	
					**August**	*0.001*	0.999	0.185	-	-	5.091%	
					**September**	0.159	0.830	*0.001*	0.618	-	5.448%	
					**October**	*0.032*	0.185	0.731	0.918	1.000	0.089%	

Table S5 continued.

**Family**	**Friedman test**					**Nemenyi pair-wise comparison P-values**					**mean monthly relative abundance**	**Boxplot of monthly relative abundance**
	**X^2**	**df**	**P**	**FDR adjusted P**		**March**	**April**	**May**	**August**	**September**		Mar April May Aug Sept Oct
**[Mogibacteriaceae]**	30.78	5	*<0.001*	*<0.001*	**March**	-	-	-	-	-	0.055%	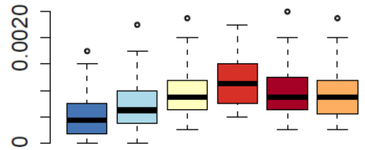
					**April**	0.283	-	-	-	-	0.074%	
					**May**	*0.006*	0.731	-	-	-	0.098%	
					**August**	*<0.001*	*0.036*	0.618	-	-	0.114%	
					**September**	*0.009*	0.793	1.000	0.546	-	0.096%	
					**October**	*0.003*	0.594	1.000	0.753	1.000	0.097%	
**Erysipelotrichaceae**	43.57	5	*<0.001*	*<0.001*	**March**	-	-	-	-	-	0.082%	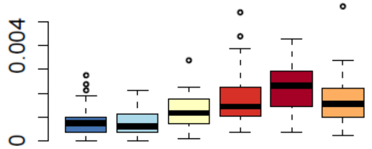
					**April**	0.999	-	-	-	-	0.080%	
					**May**	0.499	0.283	-	-	-	0.127%	
					**August**	*0.005*	*0.001*	0.475	-	-	0.181%	
					**September**	*<0.001*	*<0.001*	*0.021*	0.753	-	0.227%	
					**October**	*0.029*	*0.009*	0.793	0.996	0.429	0.172%	
**Alcaligenaceae**	47.96	5	*<0.001*	*<0.001*	**March**	-	-	-	-	-	0.591%	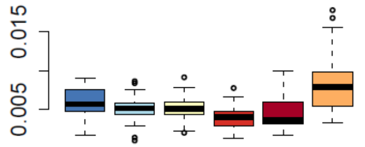
					**April**	0.594	-	-	-	-	0.506%	
					**May**	0.964	0.970	-	-	-	0.517%	
					**August**	*0.001*	0.124	*0.013*	-	-	0.393%	
					**September**	0.072	0.879	0.406	0.731	-	0.446%	
					**October**	0.248	*0.002*	*0.032*	*<0.001*	*<0.001*	0.837%	
**Oxalobacteraceae**	24.87	5	*<0.001*	*<0.001*	**March**	-	-	-	-	-	0.067%	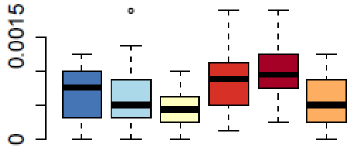
					**April**	0.984	-	-	-	-	0.061%	
					**May**	0.498	0.893	-	-	-	0.045%	
					**August**	0.879	0.475	*0.049*	-	-	0.087%	
					**September**	0.095	*0.013*	*<0.001*	0.664	-	0.100%	
					**October**	0.988	1.000	0.879	0.498	*0.015*	0.057%	

Table S5 continued.

**Family**	**Friedman test**					**Nemenyi pair-wise comparison P-values**					**mean monthly relative abundance**	**Boxplot of monthly relative abundance**
	**X^2**	**df**	**P**	**FDR adjusted P**		**March**	**April**	**May**	**August**	**September**		Mar April May Aug Sept Oct
**Desulfovibrionaceae**	45.3	5	*<0.001*	*<0.001*	**March**	-	-	-	-	-	1.156%	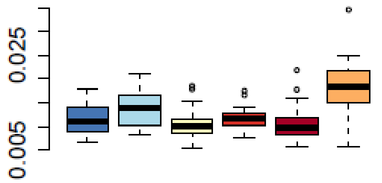
					**April**	0.452	-	-	-	-	1.356%	
					**May**	0.710	*0.015*	-	-	-	1.053%	
					**August**	1.000	0.452	0.710	-	-	1.154%	
					**September**	0.687	*0.013*	1.000	0.687	-	1.044%	
					**October**	*0.001*	0.215	*<0.001*	*0.001*	*<0.001*	1.827%	
**Campylobacteraceae**	31.34	5	*<0.001*	*<0.001*	**March**	-	-	-	-	-	0.015%	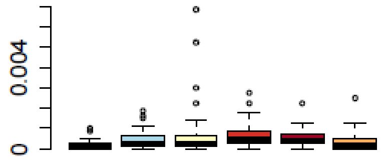
					**April**	*0.049*	-	-	-	-	0.044%	
					**May**	*0.008*	0.993	-	-	-	0.091%	
					**August**	*<0.001*	0.265	0.618	-	-	0.064%	
					**September**	*<0.001*	0.773	0.976	0.964	-	0.052%	
					**October**	0.114	1.000	0.948	0.135	0.570	0.037%	
**Helicobacteraceae**	17.69	5	*0.003*	*0.004*	**March**	-	-	-	-	-	0.007%	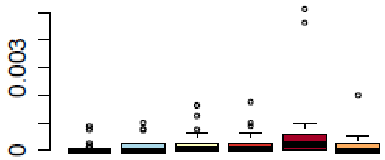
					**April**	0.687	-	-	-	-	0.014%	
					**May**	0.124	0.906	-	-	-	0.023%	
					**August**	0.546	1.000	0.964	-	-	0.022%	
					**September**	*0.016*	0.498	0.980	0.641	-	0.056%	
					**October**	0.570	1.000	0.956	1.000	0.618	0.017%	
**Succinivibrionaceae**	43.94	5	*<0.001*	*<0.001*	**March**	-	-	-	-	-	1.743%	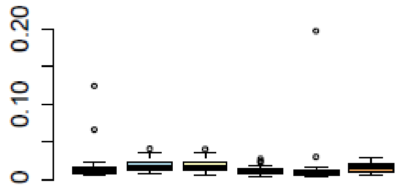
					**April**	0.215	-	-	-	-	1.798%	
					**May**	0.710	0.964	-	-	-	1.800%	
					**August**	0.363	*<0.001*	0.009	-	-	1.164%	
					**September**	*0.029*	*<0.001*	*<0.001*	0.893	-	1.578%	
					**October**	0.773	0.939	1.000	*0.013*	*<0.001*	1.640%	

Table S5 continued.

Family	Friedman test					Nemenyi pair-wise comparison P-values					mean monthly relative abundance	Boxplot of monthly relative abundance
	X^2	df	P	FDR adjusted P		March	April	May	August	September		Mar April May Aug Sept Oct
Enterobacteriaceae	25.44	5	*<0.001*	*<0.001*	**March**	-	-	-	-	-	0.004%	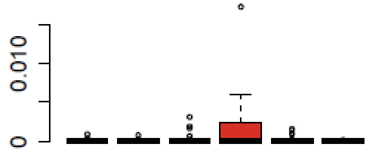
					**April**	0.995	-	-	-	-	0.005%	
					**May**	0.929	0.998	-	-	-	0.027%	
					**August**	0.095	0.302	0.570	-	-	0.157%	
					**September**	0.993	1.000	0.999	0.322	-	0.016%	
					**October**	1.000	0.980	0.863	0.060	0.976	0.001%	
Pasteurellaceae	14.36	5	*0.013*	*0.015*	**March**	-	-	-	-	-	0.009%	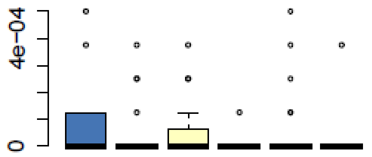
					**April**	0.960	-	-	-	-	0.004%	
					**May**	0.990	1.000	-	-	-	0.005%	
					**August**	0.430	0.910	0.790	-	-	0.000%	
					**September**	0.860	1.000	0.990	0.980	-	0.004%	
					**October**	0.450	0.920	0.810	1.000	0.980	0.001%	
**Sphaerochaetaceae**	44.14	5	*<0.001*	*<0.001*	**March**	-	-	-	-	-	1.243%	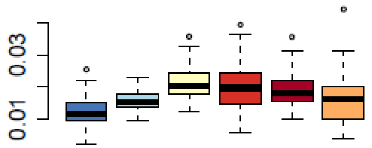
					**April**	0.146	-	-	-	-	1.547%	
					**May**	*<0.001*	*0.005*	-	-	-	2.117%	
					**August**	*0.001*	0.664	0.302	-	-	1.994%	
					**September**	*<0.001*	0.172	0.830	0.957	-	1.961%	
					**October**	0.104	1.000	*0.008*	0.753	0.231	1.602%	
**Spirochaetaceae**	23.98	5	*<0.001*	*<0.001*	**March**	-	-	-	-	-	0.625%	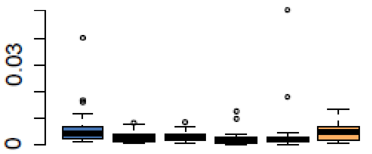
					**April**	0.215	-	-	-	-	0.298%	
					**May**	0.172	1.000	-	-	-	0.300%	
					**August**	*0.001*	0.475	0.546	-	-	0.234%	
					**September**	*0.005*	0.773	0.830	0.997	-	0.392%	
					**October**	0.956	0.731	0.664	*0.019*	0.072	0.499%	

Table S5 continued.

**Family**	**Friedman test**					**Nemenyi pair-wise comparison P-values**					**mean monthly relative abundance**	**Boxplot of monthly relative abundance**
	**X²**	**df**	**P**	**FDR adjusted P**		**March**	**April**	**May**	**August**	**September**		Mar April May Aug Sept Oct
**Dethiosulfovibrionaceae**	72.35	5	*<0.001*	*<0.001*	**March**	-	-	-	-	-	2.539%	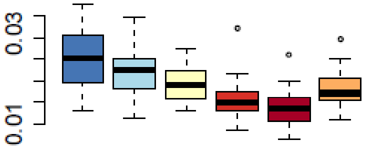
					**April**	0.363	-	-	-	-	2.173%	
					**May**	0.231	1.000	-	-	-	1.921%	
					**August**	*<0.001*	*<0.001*	*<0.001*	-	-	1.535%	
					**September**	*<0.001*	*<0.001*	*<0.001*	1.000	-	1.377%	
					**October**	*<0.001*	0.200	0.322	0.185	0.159	1.809%	
**Synergistaceae**	55.39	5	*<0.001*	*<0.001*	**March**	-	-	-	-	-	0.403%	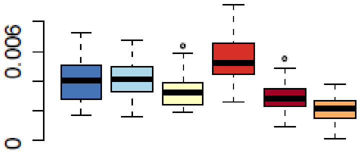
					**April**	0.999	-	-	-	-	0.414%	
					**May**	0.731	0.498	-	-	-	0.336%	
					**August**	0.080	0.185	*0.001*	-	-	0.546%	
					**September**	0.215	0.095	0.956	*<0.001*	-	0.287%	
					**October**	*0.001*	*<0.001*	0.072	*<0.001*	0.429	0.214%	
**unclass. Mollicutes**	40.14	5	*<0.001*	*<0.001*	**March**	-	-	-	-	-	0.063%	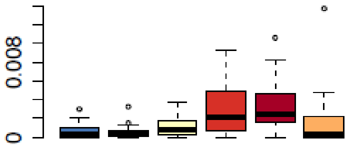
					**April**	1.000	-	-	-	-	0.054%	
					**May**	0.830	0.753	-	-	-	0.111%	
					**August**	*0.007*	*0.004*	0.215	-	-	0.284%	
					**September**	*<0.001*	*<0.001*	*0.013*	0.906	-	0.319%	
					**October**	0.964	0.929	0.999	0.087	*0.003*	0.146%	
**[Cerasicoccaceae]**	34.3	5	*<0.001*	*<0.001*	**March**	-	-	-	-	-	3.241%	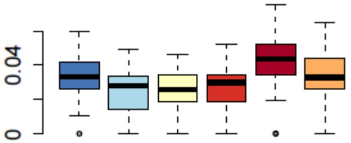
					**April**	0.406	-	-	-	-	2.467%	
					**May**	0.265	1.000	-	-	-	2.458%	
					**August**	0.753	0.995	0.970	-	-	2.708%	
					**September**	*0.060*	*<0.001*	*<0.001*	*<0.001*	-	4.143%	
					**October**	0.999	0.664	0.498	0.929	*0.019*	3.280%	

Table S6: Rho-values of Spearman rank correlations testing the association of mean monthly abundance of major microbial phyla with diet measures. FDR-adjusted q-values are shown in brackets. Sample size was 6 in all cases. Intake of fruit, leaves and flowers was calculated as mean feeding time dedicated to these items per month (in minutes per hour of total feeding time). Mean monthly intake of macronutrients was measured in percent of overall macronutrient intake, mean monthly overall energy intake as kcal / hour. Results of significant tests (FDR-corrected q-value ≤ 0.05) are printed in bold.

**Phylum**	**fruit intake**	**intake of mature leaves**	**intake of young leaves**	**intake of flowers**	**intake of TNCE***	**intake of crude protein**	**intake of fiber**	**intake of fat**	**overall energy intake**
Actinobacteria	-0.6 (q = 0.4)	0.31 (q = 0.92)	-0.26 (q = 0.71)	-0.37 (q = 0.83)	0.6 (q = 0.48)	0.03 (q = 1)	0.71 (q = 0.4)	-0.66 (q = 0.44)	-0.31 (q = 0.56)
**Bacteroidetes**	**1 (q = 0.009**)	-0.09 (q = 0.92)	-0.2 (q = 0.71)	-0.09 (q = 0.92)	-0.89 (q = 0.11)	-0.31 (q = 0.94)	-0.49 (q = 0.4)	0.71 (q = 0.44)	0.54 (q = 0.42)
Cyanobacteria	-0.31 (q = 0.63)	0.83 (q = 0.34)	-0.71 (q = 0.68)	-0.83 (q = 0.58)	-0.03 (q = 1)	-0.26 (q = 0.94)	0.66 (q = 0.4)	0.09 (q = 0.92)	-0.77 (q = 0.26)
Fibrobacteres	0.31 (q = 0.63)	-0.77 (q = 0.34)	0.2 (q = 0.71)	0.54 (q = 0.74)	-0.31 (q = 0.7)	-0.31 (q = 0.94)	-0.71 (q = 0.4)	0.37 (q = 0.71)	0.37 (q = 0.56)
**Firmicutes**	**-1 (q = 0.009)**	0.09 (q = 0.92)	0.2 (q = 0.71)	0.09 (q = 0.92)	0.89 (q = 0.11)	0.31 (q = 0.94)	0.49 (q = 0.4)	-0.71 (q = 0.44)	-0.54 (q = 42)
Proteobacteria	0.26 (q = 0.66)	-0.77 (q = 0.34)	0.83 (q = 0.58)	0.71 (q = 0.58)	0.09 (q = 1)	0.54 (q = 0.94)	-0.6 (q = 0.4)	-0.14 (q = 0.89)	0.89 (q = 0.26)
Spirochaetes	-0.54 (q = 0.43)	0.71 (q = 0.34)	-0.49 (q = 0.71)	-0.37 (q = 0.83)	0.54 (q = 0.5)	-0.26 (q = 0.94)	0.89 (q = 0.33)	-0.6 (q = 0.48)	-0.77 (q = 0.26)
**Synergistetes**	**1 (q = 0.009**)	-0.09 (q = 0.92)	-0.2 (q = 0.71)	-0.09 (q = 0.92)	-0.89 (q = 0.11)	-0.31 (q = 0.94)	-0.49 (q = 0.4)	0.71 (q = 0.44)	0.54 (q = 0.42)
Tenericutes	-0.89 (q = 0.08)	0.26 (q = 0.92)	-0.2 (q = 0.71)	-0.09 (q = 0.92)	0.66 (q = 0.44)	-0.14 (q = 1)	0.54 (q = 0.4)	-0.48 (q = 0.59)	-0.83 (q = 0.26)
Verrucomicrobia	-0.6 (q = 0.4)	-0.54 (q = 0.59)	0.43 (q = 0.71)	0.66 (q = 0.58)	0.43 (q = 0.6)	0.03 (q = 1)	-0.31 (q = 0.56)	-0.14 (q = 0.89)	-0.31 (q = 0.56)

*TNCE = non-structural carbohydrates

Table S7: Rho-values of Spearman rank correlations testing the association of mean monthly abundance of major microbial families with diet measures. FDR-adjusted q-values are shown in brackets. Sample size was 6 in all cases. Intake of fruit, leaves and flowers was calculated as mean feeding time dedicated to these items per month (in minutes per hour of total feeding time). Mean monthly intake of macronutrients was measured in percent of overall macronutrient intake, mean monthly overall energy intake as kcal / hour. Results of significant tests (FDR-corrected q-value ≤ 0.05) are printed in bold.

**Family**	**fruit intake**	**intake of mature leaves**	**intake of young leaves**	**intake of flowers**	**intake of TNCE***	**intake of crude protein**	**intake of fiber**	**intake of fat**	**overall energy intake**
Coriobacteriaceae	-0.6 (q = 0.6)	0.31 (q = 0.9)	-0.26 (q = 0.85)	-0.37 (q = 0.88)	0.6 (q = 0.69)	0.03 (q = 1)	0.71 (q = 0.42)	-0.66 (q = 0.69)	-0.31 (q = 0.77)
unclass. Actinobacteria	-0.14 (q = 0.85)	0.26 (q = 0.94)	-0.31 (q = 0.85)	-0.09 (q = 0.94)	0.43 (q = 0.74)	-0.31 (q = 1)	0.60 (q = 0.53)	-0.66 (q = 0.69)	-0.14 (q = 0.87)
unclass. Bacteroidales	0.77 (q = 0.38)	-0.2 (q = 0.98)	0.09 (q = 0.92)	0.31 (q = 0.91)	-0.49 (q = 0.69)	-0.2 (q = 1)	-0.43 (q = 0.57)	0.31 (q = 0.83)	0.49 (q = 0.73)
Bacteroidales f. BS11	-0.37 (q = 0.72)	0.54 (q = 0.55)	0.09 (q = 0.92)	-0.26 (q = 0.91)	0.49 (q = 0.69)	0.49 (q = 1)	0.60 (q = 0.53)	-0.54 (q = 0.73)	-0.2 (q = 0.83)
Bacteroidaceae	0.89 (q = 0.15)	0.03 (q = 1)	-0.37 (q = 0.85)	-0.26 (q = 0.91)	-1.00 (q = 0.1)	-0.43 (q = 1)	-0.49 (q = 0.57)	0.94 (q = 0.62)	0.26 (q = 0.83)
Porphyromonadaceae	0.54 (q = 0.6)	0.71 (q = 0.4)	-0.54 (q = 0.85)	-0.77 (q = 0.38)	-0.49 (q = 0.69)	-0.09 (q = 1)	0.37 (q = 0.63)	0.26 (q = 0.87)	0.09 (q = 0.97)
Prevotellaceae	0.89 (q = 0.15)	0.14 (q = 1)	-0.2 (q = 0.85)	-0.31 (q = 0.91)	-0.89 (q = 0.25)	-0.09 (q = 1)	-0.37 (q = 0.63)	0.77 (q = 0.69)	0.43 (q = 0.77)
Bacteroidales f. RF16	-0.14 (q = 0.85)	-0.71 (q = 0.4)	0.6 (q = 0.85)	0.94 (q = 0.21)	0.37 (q = 0.8)	0.03 (q = 1)	-0.43 (q = 0.57)	-0.31 (q = 0.83)	0.2 (q = 0.83)
Bacteroidales f. S24-7	0.71 (q = 0.46)	0.54 (q = 0.55)	-0.43 (q = 0.85)	-0.54 (q = 0.62)	-0.60 (q = 0.69)	-0.14 (q = 1)	0.14 (q = 0.9)	0.37 (q = 0.83)	0.2 (q = 0.83)
[Paraprevotellaceae]	0.43 (q = 0.67)	-0.66 (q = 0.4)	0.43 (q = 0.85)	0.54 (q = 0.62)	-0.54 (q = 0.69)	0.03 (q = 1)	-0.94 (q = 0.15)	0.71 (q = 0.69)	0.37 (q = 0.77)
unclass. Cyanobacteria YS2	-0.31 (q = 0.72)	0.83 (q = 0.31)	-0.71 (q = 0.85)	-0.83 (q = 0.31)	-0.03 (q = 1)	-0.26 (q = 1)	0.66 (q = 0.5)	0.09 (q = 1)	-0.77 (q = 0.64)
unclass. Streptophyta	0.49 (q = 0.6)	0.09 (q = 1)	0.14 (q = 0.9)	-0.09 (q = 0.94)	-0.09 (q = 0.97)	0.31 (q = 1)	0.09 (q = 0.95)	-0.20 (q = 0.88)	0.66 (q = 0.64)
Fibrobacteraceae	0.31 (q = 0.72)	-0.77 (q = 0.38)	0.2 (q = 0.85)	0.54 (q = 0.62)	-0.31 (q = 0.87)	-0.31 (q = 1)	-0.71 (q = 0.42)	0.37 (q = 0.84)	0.37 (q = 0.77)

Table S7 continued.

**Family**	**fruit intake**	**intake of mature leaves**	**intake of young leaves**	**intake of flowers**	**intake of TNCE***	**intake of crude protein**	**intake of fiber**	**intake of fat**	**overall energy intake**
Streptococcaceae	-0.14 (q = 0.85)	0.83 (q = 0.31)	-0.6 (q = 0.85)	-0.94 (q = 0.21)	0.03 (q = 1)	0.03 (q = 1)	0.77 (q = 0.38)	-0.14 (q = 0.93)	-0.31 (q = 0.77)
unclass. Clostridiales	-1 (q = 0.1)	0.09 (q = 1)	0.2 (q = 0.85)	0.09 (q = 0.94)	0.89 (q = 0.25)	0.31 (q = 1)	0.49 (q = 0.57)	-0.71 (q = 0.69)	-0.54 (q = 0.65)
Clostridiaceae	-0.94 (q = 0.15)	-0.09 (q = 1)	0.31 (q = 0.85)	0.2 (q = 0.91)	0.94 (q = 0.21)	0.37 (q = 1)	0.43 (q = 0.57)	-0.83 (q = 0.69)	-0.31 (q = 0.77)
Eubacteriaceae	-0.54 (q = 0.6)	0.77 (q = 0.38)	-0.54 (q = 0.85)	-0.77 (q = 0.38)	0.26 (q = 0.9)	-0.03 (q = 1)	0.77 (q = 0.38)	-0.20 (q = 0.88)	-0.71 (q = 0.64)
**Lachnospiraceae**	-0.49 (q = 0.6)	0.83 (q = 0.31)	-0.54 (q = 0.85)	-0.66 (q = 0.46)	0.49 (q = 0.69)	-0.09 (q = 1)	**1 (q = 0.05)**	-0.60 (q = 0.69)	-0.6 (q = 0.64)
Ruminococcaceae	-0.94 (q = 0.15)	-0.09 (q = 1)	0.31 (q = 0.85)	0.2 (q = 0.91)	0.94 (q= 0.21)	0.37 (q = 1)	0.43 (q = 0.57)	-0.83 (q = 0.69)	-0.31 (q = 0.77)
Veillonellaceae	0.09 (q = 0.92)	-0.89 (q = 0.31)	0.54 (q = 0.85)	0.89 (q = 0.25)	-0.09 (q = 0.97)	-0.09 (q = 1)	-0.83 (q = 0.31)	0.26 (q = 0.87)	0.26 (q = 0.83)
[Mogibacteriaceae]	-0.49 (q = 0.6)	0.6 (q = 0.53)	-0.49 (q = 0.85)	-0.6 (q = 0.6)	0.49 (q = 0.69)	-0.09 (q = 1)	0.89 (q = 0.25)	-0.60 (q = 0.69)	-0.43 (q = 0.77)
Erysipelotrichaceae	-0.89 (q = 0.15)	0.26 (q = 0.94)	-0.2 (q = 0.85)	-0.09 (q = 0.94)	0.66 (q = 0.65)	-0.14 (q = 1)	0.54 (q = 0.55)	-0.49 (q = 0.73)	-0.83 (q = 0.64)
Alcaligenaceae	0.26 (q = 0.79)	-0.66 (q = 0.4)	0.54 (q = 0.85)	0.77 (q = 0.38)	0.14 (q = 0.97)	0.09 (q = 1)	-0.43 (q = 0.57)	-0.26 (q = 0.87)	0.6 (q = 0.64)
Oxalobacteraceae	0.26 (q = 0.72)	0.03 (q = 1)	-0.2 (q = 0.85)	-0.09 (q = 0.94)	-0.14 (q = 0.97)	-0.26 (q = 1)	-0.14 (q = 0.9)	0.43 (q = 0.82)	-0.6 (q = 0.64)
Desulfovibrionaceae	-0.31 (q = 0.72)	-0.77 (q = 0.38)	0.6 (q = 0.85)	0.49 (q = 0.69)	-0.09 (q = 0.97)	0.37 (q = 1)	-0.60 (q = 0.53)	0.03 (q = 1)	0.89 (q = 0.64)
Campylobacteraceae	0.31 (q = 0.72)	0.94 (q = 0.31)	-0.6 (q = 0.85)	-0.83 (q = 0.31)	0.26 (q = 0.9)	-0.03 (q = 1)	0.94 (q = 0.15)	-0.37 (q = 0.83)	-0.54 (q = 0.65)
Helicobacteraceae	-0.77 (q = 0.38)	0.66 (q = 0.4)	-0.26 (q = 0.85)	-0.37 (q = 0.88)	0.66 (q = 0.65)	0.09 (q = 1)	0.83 (q = 0.31)	-0.60 (q = 0.69)	-0.77 (q = 0.64)
Succinivibrionaceae	0.6 (q = 0.6)	0.09 (q = 1)	0.14 (q = 0.9)	0.03 (q = 1)	-0.26 (q = 0.9)	0.2 (q = 1)	-0.09 (q = 0.94)	0.03 (q = 1)	0.54 (q = 0.65)

Table S7 continued.

**Family**	**fruit intake**	**intake of mature leaves**	**intake of young leaves**	**intake of flowers**	**intake of TNCE***	**intake of crude protein**	**intake of fiber**	**intake of fat**	**overall energy intake**
Enterobacteriaceae	-0.14 (q = 0.85)	0.94 (q = 0.31)	-0.83 (q = 0.85)	-0.94 (q = 0.21)	-0.09 (q = 0.97)	-0.31 (q = 1)	0.77 (q = 0.38)	0.03 (q = 1)	-0.66 (q = 0.64)
Pasteurellaceae	0.49 (q = 0.6)	0.03 (q = 1)	-0.09 (q = 0.9)	0.26 (q = 0.91)	-0.37 (q = 0.8)	-0.37 (q = 1)	-0.26 (q = 0.79)	0.31 (q = 0.83)	-0.03 (q = 1)
**Sphaerochaetaceae**	-0.49 (q = 0.6)	0.83 (q = 0.31)	-0.54 (q = 0.85)	-0.66 (q = 0.46)	0.49 (q = 0.69)	-0.09 (q = 1)	**1.00 (q = 0.05)**	-0.60 (q = 0.69)	-0.6 (q = 0.64)
Spirochaetaceae	0.09 (q = 0.92)	-0.66 (q = 0.4)	0.43 (q = 0.85)	0.89 (q = 0.25)	0.09 (q = 0.97)	-0.2 (q = 1)	-0.54 (q = 0.55)	-0.03 (q = 1)	0.14 (q = 0.87)
Dethiosulfovibrionaceae	0.94 (q = 0.15)	-0.31 (q = 0.91)	0.09 (q = 0.9)	0.2 (q = 0.91)	-0.71 (q = 0.65)	-0.14 (q = 1)	-0.60 (q = 0.53)	0.54 (q = 0.73)	0.71 (q = 0.64)
Synergistaceae	0.54 (q = 0.6)	0.37 (q = 0.88)	-0.6 (q = 0.85)	-0.71 (q = 0.46)	-0.77 (q = 0.64)	-0.31 (q = 1)	-0.03 (q = 1)	0.71 (q = 0.69)	0.03 (q = 1)
unclass. Mollicutes RF39	-0.89 (q = 0.15)	0.26 (q = 0.94)	-0.2 (q = 0.85)	-0.09 (q = 0.94)	0.66 (q = 0.65)	-0.14 (q = 1)	0.54 (q = 0.55)	-0.49 (q = 0.73)	-0.83 (q = 0.64)
[Cerasicoccaceae]	-0.6 (q = 0.6)	-0.54 (q = 0.55)	0.43 (q = 0.85)	0.66 (q = 0.46)	0.43 (q = 0.74)	0.03 (q = 1)	-0.31 (q = 0.7)	-0.14 (q = 0.93)	-0.31 (q = 0.77)

Table S8: Results of genus-level analyses of monthly abundance. Genera for which significant differences in monthly abundance were found are printed in bold. Significant P-values (< 0.05) are printed in bold. Only tests for genera with q-value ≤ 0.05 in the Friedman test are shown.

	Friedman test			Nemenyi multiple comparison P-values						
**Genus**	**X²**	**df**	**FDR adjusted P (q)**		**March**	**April**	**May**	**August**	**September**	**mean monthly relative abundance**
**unclassified Coriobacteriaceae**	19.4	5	**0.012**	**March**	-	-	-	-	-	0.0199
				**April**	**0.008**	-	-	-	-	0.0271
				**May**	**0.01**	1	-	-	-	0.0291
				**August**	0.641	0.406	0.452	-	-	0.0220
				**September**	0.384	0.664	0.71	0.999	-	0.0227
				**October**	0.984	0.066	0.08	0.956	0.812	0.0226
**Slackia**	29.12	5	**<0.001**	**March**	-	-	-	-	-	0.0008
				**April**	**0.006**	-	-	-	-	0.0028
				**May**	**<0.001**	0.984	-	-	-	0.0025
				**August**	**0.044**	0.991	0.793	-	-	0.0024
				**September**	0.200	0.830	0.406	0.991	-	0.0018
				**October**	**<0.001**	0.830	0.995	0.452	0.146	0.0030
**Parabacteroides**	17.26	5	**0.027**	**March**	-	-	-	-	-	0.0011
				**April**	0.976	-	-	-	-	0.0010
				**May**	0.995	1.000	-	-	-	0.0009
				**August**	1.000	0.991	0.999	-	-	0.0010
				**September**	0.997	0.830	0.918	0.991	-	0.0013
				**October**	0.080	0.384	0.265	0.114	**0.021**	0.0005
**Prevotella**	45.13	5	**<0.001**	**March**	-	-	-	-	-	0.0599
				**April**	0.999	-	-	-	-	0.0585
				**May**	1.000	1.000	-	-	-	0.0561
				**August**	**0.009**	**0.029**	**0.015**	-	-	0.0338
				**September**	**0.006**	**0.021**	**0.010**	1.000	-	0.0287
				**October**	**<0.001**	**<0.001**	**<0.001**	0.710	0.773	0.0257

Table S8 continued.

Genus	X^2	df	FDR adjusted P (q)		March	April	May	August	September	mean monthly relative abundance
			**Friedman test**		**Nemenyi multiple comparison P-values**					
**unclassified Cyanobacteria YS2**	17.06	5	**0.029**	**March**	-	-	-	-	-	0.0695
				**April**	0.998	-	-	-	-	0.0720
				**May**	0.999	1.000	-	-	-	0.0640
				**August**	0.087	0.231	0.200	-	-	0.0943
				**September**	0.996	0.939	0.957	**0.021**	-	0.0656
				**October**	0.830	0.570	0.618	**0.002**	0.980	0.0517
**unclassified Streptophyta**	76.21	5	**<0.001**	**March**	-	-	-	-	-	0.0004
				**April**	**0.004**	-	-	-	-	0.0016
				**May**	**<0.001**	0.664	-	-	-	0.0037
				**August**	0.948	**<0.001**	**<0.001**	-	-	0.0002
				**September**	0.918	**<0.001**	**<0.001**	1.000	-	0.0002
				**October**	0.964	0.054	**<0.001**	0.522	0.452	0.0006
**unclassified Clostridiales**	32.18	5	**<0.001**	**March**	-	-	-	-	-	0.2193
				**April**	1.000	-	-	-	-	0.2217
				**May**	0.753	0.893	-	-	-	0.2403
				**August**	0.918	0.980	0.999	-	-	0.2347
				**September**	**<0.001**	**<0.001**	**0.010**	**0.003**	-	0.2749
				**October**	**0.040**	0.087	0.618	0.385	0.475	0.2548
**unclassified Lachnospiraceae**	59.14	5	**<0.001**	**March**	-	-	-	-	-	0.0652
				**April**	**<0.001**	-	-	-	-	0.1185
				**May**	**<0.001**	0.710	-	-	-	0.1348
				**August**	**<0.001**	1.000	0.830	-	-	0.1220
				**September**	0.104	0.087	**0.001**	**0.049**	-	0.0907
				**October**	**0.049**	0.172	**0.002**	0.104	1.000	0.1008

Table S8 continued.

Genus	X²	df	FDR adjusted P (q)		March	April	May	August	September	mean monthly relative abundance
	**Friedman test**				**Nemenyi multiple comparison P-values**					
Anaerostipes	18.29	5	**0.018**	**March**	-	-	-	-	-	0.0015
				**April**	0.995	-	-	-	-	0.0003
				**May**	0.847	0.988	-	-	-	0.0002
				**August**	0.993	0.879	0.498	-	-	0.0044
				**September**	0.687	0.342	0.087	0.948	-	0.0073
				**October**	0.976	0.793	0.384	1.000	0.980	0.0047
**Butyrivibrio**	36.34	5	**<0.001**	**March**	-	-	-	-	-	0.0695
				**April**	0.231	-	-	-	-	0.0895
				**May**	**<0.001**	0.087	-	-	-	0.1018
				**August**	1.000	0.302	**<0.001**	-	-	0.0752
				**September**	**0.023**	0.948	0.498	0.036	-	0.0914
				**October**	0.964	0.731	**0.001**	0.984	0.200	0.0739
Anaerofilum	16.38	5	**0.036**	**March**	-	-	-	-	-	0.0001
				**April**	0.550	-	-	-	-	0.0003
				**May**	0.550	1.000	-	-	-	0.0003
				**August**	1.000	0.730	0.730	-	-	0.0001
				**September**	1.000	0.770	0.770	1.000	-	0.0002
				**October**	0.230	0.990	0.990	0.380	0.430	0.0003
**Oscillospira**	38.25	5	**<0.001**	**March**	-	-	-	-	-	0.0120
				**April**	**0.032**	-	-	-	-	0.0165
				**May**	0.080	1.000	-	-	-	0.0165
				**August**	**<0.001**	**0.044**	**0.016**	-	-	0.0229
				**September**	**0.002**	0.976	0.893	0.265	-	0.0198
				**October**	**<0.001**	0.847	0.664	0.522	0.998	0.0211

Table S8 continued.

	Friedman test				Nemenyi multiple comparison P-values					
**Genus**	**X²**	**df**	**FDR adjusted P (q)**		**March**	**April**	**May**	**August**	**September**	**mean monthly relative abundance**
**Papillibacter**	26.18	5	**<0.001**	**March**	-	-	-	-	-	0.0011
				**April**	0.664	-	-	-	-	0.0015
				**May**	**0.019**	0.546	-	-	-	0.0021
				**August**	1.000	0.812	**0.040**	-	-	0.0010
				**September**	0.991	0.948	0.104	0.999	-	0.0011
				**October**	0.687	**0.036**	**<0.001**	0.522	0.302	0.0007
Ruminococcus	22.34	5	**0.004**	**March**	-	-	-	-	-	0.0014
				**April**	0.283	-	-	-	-	0.0044
				**May**	0.066	0.988	-	-	-	0.0047
				**August**	1.000	0.342	0.087	-	-	0.0010
				**September**	0.114	0.998	1.000	0.146	-	0.0048
				**October**	0.104	0.997	1.000	0.135	1.000	0.0097
**unclass. Veillonellaceae**	20.95	5	**0.007**	**March**	-	-	-	-	-	0.0036
				**April**	0.710	-	-	-	-	0.0025
				**May**	**0.004**	0.248	-	-	-	0.0017
				**August**	**0.003**	0.215	1.000	-	-	0.0020
				**September**	0.231	0.970	0.731	0.687	-	0.0024
				**October**	0.664	1.000	0.283	0.248	0.980	0.0029
**Phascolarctobacterium**	63.52	5	**<0.001**	**March**	-	-	-	-	-	0.2173
				**April**	**0.001**	-	-	-	-	0.1409
				**May**	**<0.001**	**0.016**	-	-	-	0.0996
				**August**	**<0.001**	0.406	0.773	-	-	0.1242
				**September**	**<0.001**	0.988	0.104	0.812	-	0.1280
				**October**	**0.036**	0.893	**<0.001**	**0.036**	0.522	0.1490

Table S8 continued.

Genus	X²	df	FDR adjusted P (q)		March	April	May	August	September	mean monthly relative abundance
			**Friedman test**		**Nemenyi multiple comparison P-values**					
**Coprobacillus**	34.46	5	**<0.001**	**March**	-	-	-	-	-	0.0017
				**April**	0.970	-	-	-	-	0.0015
				**May**	0.773	0.283	-	-	-	0.0024
				**August**	**0.026**	**0.002**	0.499	-	-	0.0032
				**September**	**0.001**	**<0.001**	0.087	0.948	-	0.0039
				**October**	0.200	**0.026**	0.929	0.970	0.546	0.0032
Oenothera	18.7	5	**0.016**	**March**	-	-	-	-	-	0
				**April**	0.990	-	-	-	-	4.84E-05
				**May**	0.660	0.960	-	-	-	0.0001
				**August**	1.000	0.990	0.660	-	-	0
				**September**	1.000	0.990	0.660	1.000	-	0
				**October**	0.890	1.000	1.000	0.890	0.890	0.0001
**Sutterella**	27.82	5	**<0.001**	**March**	-	-	-	-	-	0.0016
				**April**	1.000	-	-	-	-	0.0014
				**May**	0.812	0.864	-	-	-	0.0011
				**August**	**0.001**	**0.001**	0.066	-	-	0.0006
				**September**	0.135	0.172	0.830	0.641	-	0.0008
				**October**	0.995	0.998	0.980	**0.007**	0.385	0.0015
**Oxalobacter**	16.77	5	**0.031**	**March**	-	-	-	-	-	0.0020
				**April**	1.000	-	-	-	-	0.0016
				**May**	0.687	0.830	-	-	-	0.0013
				**August**	1.000	0.996	0.522	-	-	0.0020
				**September**	0.248	0.146	**0.004**	0.385	-	0.0025
				**October**	0.939	0.984	0.995	0.847	**0.023**	0.0015

Table S8 continued.

	Friedman test				Nemenyi multiple comparison P-values					
**Genus**	**X²**	**df**	**FDR adjusted P (q)**		**March**	**April**	**May**	**August**	**September**	**mean monthly relative abundance**
**unclass. Desulfovibrionaceae**	55.89	5	**<0.001**	**March**	-	-	-	-	-	0.0069
				**April**	1.000	-	-	-	-	0.0074
				**May**	0.200	0.146	-	-	-	0.0050
				**August**	0.980	0.957	0.618	-	-	0.0070
				**September**	**0.049**	**0.032**	0.993	0.265	-	0.0058
				**October**	**0.002**	**0.004**	**<0.001**	**<0.001**	**<0.001**	0.0158
**Bilophila**	48.31	5	**<0.001**	**March**	-	-	-	-	-	0.0140
				**April**	0.976	-	-	-	-	0.0151
				**May**	0.159	0.021	-	-	-	0.0104
				**August**	0.710	0.248	0.929	-	-	0.0115
				**September**	**0.021**	**0.001**	0.976	0.522	-	0.0099
				**October**	0.060	0.322	**<0.001**	**<0.001**	**<0.001**	0.0203
**Desulfovibrio**	46.44	5	**<0.001**	**March**	-	-	-	-	-	0.0078
				**April**	1.000	-	-	-	-	0.0080
				**May**	0.498	0.618	-	-	-	0.0066
				**August**	**<0.001**	**0.001**	0.146	-	-	0.0045
				**September**	**<0.001**	**<0.001**	**0.013**	0.956	-	0.0041
				**October**	**0.004**	**0.007**	0.406	0.995	0.731	0.0048
**Campylobacter**	24.48	5	**0.002**	**March**	-	-	-	-	-	0.0004
				**April**	**0.044**	-	-	-	-	0.0013
				**May**	0.054	1.000	-	-	-	0.0023
				**August**	**0.001**	0.847	0.812	-	-	0.0017
				**September**	0.080	1.000	1.000	0.731	-	0.0010
				**October**	0.906	0.429	0.475	0.029	0.570	0.0008

Table S8 continued.

Genus	X^2	df	FDR adjusted P (q)		March	April	May	August	September	mean monthly relative abundance
	Friedman test				Nemenyi multiple comparison P-values					
unclass. Enterobacteriaceae	20.24	5	**0.01**	**March**	-	-	-	-	-	0.0001
				**April**	1.000	-	-	-	-	0.0001
				**May**	0.880	0.980	-	-	-	0.0007
				**August**	0.220	0.410	0.860	-	-	0.0035
				**September**	0.960	1.000	1.000	0.710	-	0.0003
				**October**	1.000	1.000	0.860	0.200	0.960	3.13E-05
Mannheimia	19.88	5	**0.011**	**March**	-	-	-	-	-	1.88E-04
				**April**	0.940	-	-	-	-	1.61E-05
				**May**	1.000	0.920	-	-	-	1.07E-04
				**August**	0.860	1.000	0.830	-	-	0
				**September**	0.860	1.000	0.830	1.000	-	1.56E-05
				**October**	0.860	1.000	0.830	1.000	1.000	1.56E-05
**unclass. Mollicutes RF39**	47.73	5	**<0.001**	**March**	-	-	-	-	-	0.0019
				**April**	0.893	-	-	-	-	0.0012
				**May**	0.929	0.322	-	-	-	0.0029
				**August**	**0.044**	**0.001**	0.385	-	-	0.0074
				**September**	**0.001**	**<0.001**	**0.032**	0.893	-	0.0075
				**October**	1.000	0.929	0.893	**0.032**	**0.001**	0.0032
**unclass. Opitutae [Cerasicoccaceae]**	22.22	5	**0.005**	**March**	-	-	-	-	-	0.0971
				**April**	0.114	-	-	-	-	0.0689
				**May**	0.060	1.000	-	-	-	0.0662
				**August**	**0.040**	0.999	1.000	-	-	0.0672
				**September**	0.999	**0.044**	**0.021**	**0.013**	-	0.0990
				**October**	0.248	0.999	0.991	0.976	0.114	0.0769

Table S9: Rho-values of Spearman rank correlations testing the association of mean monthly abundance of known microbial genera with diet measures. FDR-adjusted q-values are shown in brackets. Sample size was 6 in all cases. Intake of fruit, leaves and flowers was calculated as mean feeding time dedicated to these items per month (in minutes per hour of total feeding time). Mean monthly intake of macronutrients was measured in percent of overall macronutrient intake, mean monthly overall energy intake as kcal / hour. No significant correlations ($q \leq 0.05$) were found.

**Genus**	**fruit intake**	**intake of mature leaves**	**intake of young leaves**	**intake of flowers**	**intake of TNCE**	**intake of crude protein**	**intake of fiber**	**intake of fat**	**overall energy intake**
[Methanomassiliicoccaceae] vadinCA11	-0.12 (q = 0.91)	0.9 (q = 0.51)	-0.87 (q = 0.67)	-0.7 (q = 0.57)	0.03 (q = 0.97)	-0.58 (q = 0.89)	0.81 (q = 0.52)	-0.14 (q = 0.9)	-0.75 (q = 0.69)
uncalss Acidobacteria-6	0.39 (q = 0.9)	-0.13 (q = 0.91)	0.39 (q = 0.67)	-0.13 (q = 0.88)	-0.39 (q = 0.93)	0.65 (q = 0.72)	-0.39 (q = 0.7)	0.39 (q = 0.8)	0.65 (q = 0.69)
unclass. Acidobacteria S035	0.13 (q = 0.9)	0.65 (q = 0.51)	-0.39 (q = 0.67)	-0.39 (q = 0.73)	0.13 (q = 0.94)	-0.13 (q = 0.89)	0.65 (q = 0.52)	-0.39 (q = 0.8)	-0.13 (q = 0.88)
unclass. [Chloracidobacteria] 11-24	0.39 (q = 0.9)	-0.13 (q = 0.91)	0.39 (q = 0.67)	-0.13 (q = 0.88)	-0.39 (q = 0.93)	0.65 (q = 0.72)	-0.39 (q = 0.7)	0.39 (q = 0.8)	0.65 (q = 0.69)
unclass. [Chloracidobacteria] RB41	0.39 (q = 0.9)	-0.13 (q = 0.91)	0.39 (q = 0.67)	-0.13 (q = 0.88)	-0.39 (q = 0.93)	0.65 (q = 0.72)	-0.39 (q = 0.7)	0.39 (q = 0.8)	0.65 (q = 0.69)
Varibaculum	0.28 (q = 0.9)	0 (q = 1)	-0.06 (q = 0.94)	0.25 (q = 0.88)	0.12 (q = 0.95)	-0.28 (q = 0.89)	0.15 (q = 0.87)	-0.37 (q = 0.84)	0.19 (q = 0.88)
Corynebacterium	0.64 (q = 0.72)	0.12 (q = 0.93)	-0.03 (q = 0.97)	0.09 (q = 0.94)	-0.35 (q = 0.94)	-0.12 (q = 0.9)	-0.12 (q = 0.88)	0.14 (q = 0.9)	0.32 (q = 0.88)
Cryptosporangium	0.65 (q = 0.72)	-0.39 (q = 0.7)	-0.13 (q = 0.88)	0.39 (q = 0.73)	-0.65 (q = 0.8)	-0.65 (q = 0.72)	-0.65 (q = 0.52)	0.65 (q = 0.8)	0.13 (q = 0.88)
Knoellia	0.13 (q = 0.9)	0.65 (q = 0.51)	-0.39 (q = 0.67)	-0.39 (q = 0.73)	0.13 (q = 0.94)	-0.13 (q = 0.89)	0.65 (q = 0.52)	-0.39 (q = 0.8)	-0.13 (q = 0.88)
unclass. Jonesiaceae	-0.39 (q = 0.9)	-0.65 (q = 0.51)	0.65 (q = 0.67)	0.65 (q = 0.57)	0.65 (q = 0.8)	0.39 (q = 0.89)	-0.13 (q = 0.87)	-0.65 (q = 0.8)	0.39 (q = 0.86)
Kineococcus	0.39 (q = 0.9)	-0.13 (q = 0.91)	0.39 (q = 0.67)	-0.13 (q = 0.88)	-0.39 (q = 0.93)	0.65 (q = 0.72)	-0.39 (q = 0.7)	0.39 (q = 0.8)	0.65 (q = 0.69)

Table S9 continued.

**Genus**	**fruit intake**	**intake of mature leaves**	**intake of young leaves**	**intake of flowers**	**intake of TNCE**	**intake of crude protein**	**intake of fiber**	**intake of fat**	**overall energy intake**
unclass. Microbacteriaceae	0.13 (q = 0.9)	0.65 (q = 0.51)	-0.39 (q = 0.67)	-0.39 (q = 0.73)	0.13 (q = 0.94)	-0.13 (q = 0.89)	0.65 (q = 0.52)	-0.39 (q = 0.8)	-0.13 (q = 0.88)
Clavibacter	0.13 (q = 0.9)	0.65 (q = 0.51)	-0.39 (q = 0.67)	-0.39 (q = 0.73)	0.13 (q = 0.94)	-0.13 (q = 0.89)	0.65 (q = 0.52)	-0.39 (q = 0.8)	-0.13 (q = 0.88)
Curtobacterium	0.39 (q = 0.9)	-0.13 (q = 0.91)	0.39 (q = 0.67)	-0.13 (q = 0.88)	-0.39 (q = 0.93)	0.65 (q = 0.72)	-0.39 (q = 0.7)	0.39 (q = 0.8)	0.65 (q = 0.69)
Arthrobacter	0.39 (q = 0.9)	-0.13 (q = 0.91)	0.39 (q = 0.67)	-0.13 (q = 0.88)	-0.39 (q = 0.93)	0.65 (q = 0.72)	-0.39 (q = 0.7)	0.39 (q = 0.8)	0.65 (q = 0.69)
Microbispora	-0.65 (q = 0.72)	0.13 (q = 0.91)	0.13 (q = 0.88)	0.13 (q = 0.88)	0.39 (q = 0.93)	0.13 (q = 0.89)	0.13 (q = 0.87)	-0.13 (q = 0.9)	-0.65 (q = 0.69)
unclass. Micromonosporaceae	0.39 (q = 0.9)	-0.13 (q = 0.91)	0.39 (q = 0.67)	-0.13 (q = 0.88)	-0.39 (q = 0.93)	0.65 (q = 0.72)	-0.39 (q = 0.7)	0.39 (q = 0.8)	0.65 (q = 0.69)
Actinoplanes	0.27 (q = 0.9)	0.14 (q = 0.91)	-0.07 (q = 0.94)	-0.54 (q = 0.73)	-0.44 (q = 0.93)	0.34 (q = 0.89)	-0.1 (q = 0.89)	0.44 (q = 0.8)	0.34 (q = 0.88)
unclass. Nocardioidaceae	0.13 (q = 0.9)	0.65 (q = 0.51)	-0.39 (q = 0.67)	-0.39 (q = 0.73)	0.13 (q = 0.94)	-0.13 (q = 0.89)	0.65 (q = 0.52)	-0.39 (q = 0.8)	-0.13 (q = 0.88)
Propionibacterium	0.27 (q = 0.9)	0.14 (q = 0.91)	-0.07 (q = 0.94)	-0.54 (q = 0.73)	-0.44 (q = 0.93)	0.34 (q = 0.89)	-0.1 (q = 0.89)	0.44 (q = 0.8)	0.34 (q = 0.88)
Actinomycetospora	0.39 (q = 0.9)	-0.13 (q = 0.91)	0.39 (q = 0.67)	-0.13 (q = 0.88)	-0.39 (q = 0.93)	0.65 (q = 0.72)	-0.39 (q = 0.7)	0.39 (q = 0.8)	0.65 (q = 0.69)
Pseudonocardia	0.41 (q = 0.9)	0 (q = 1)	-0.62 (q = 0.67)	-0.21 (q = 0.88)	-0.62 (q = 0.83)	-0.83 (q = 0.72)	-0.21 (q = 0.87)	0.62 (q = 0.8)	-0.21 (q = 0.88)
Streptomyces	0.39 (q = 0.9)	-0.13 (q = 0.91)	0.39 (q = 0.67)	-0.13 (q = 0.88)	-0.39 (q = 0.93)	0.65 (q = 0.72)	-0.39 (q = 0.7)	0.39 (q = 0.8)	0.65 (q = 0.69)
Auritibacter	0.21 (q = 0.9)	-0.83 (q = 0.51)	0.41 (q = 0.67)	0.83 (q = 0.57)	0 (q = 1)	-0.21 (q = 0.89)	-0.62 (q = 0.59)	0 (q = 1)	0.41 (q = 0.86)
Bifidobacterium	-0.07 (q = 0.96)	-0.03 (q = 0.99)	0.44 (q = 0.67)	-0.03 (q = 0.96)	-0.1 (q = 0.95)	0.68 (q = 0.72)	-0.27 (q = 0.84)	0.27 (q = 0.9)	0.17 (q = 0.88)

Table S9 continued.

Genus	fruit intake	intake of mature leaves	intake of young leaves	intake of flowers	intake of TNCE	intake of crude protein	intake of fiber	intake of fat	overall energy intake
unclass. Coriobacteriaceae	-0.14 (q = 0.9)	0.49 (q = 0.7)	0.14 (q = 0.88)	-0.31 (q = 0.88)	0.31 (q = 0.94)	0.6 (q = 0.89)	0.49 (q = 0.7)	-0.43 (q = 0.8)	0.09 (q = 0.95)
Adlercreutzia	0.03 (q = 1)	0.43 (q = 0.7)	-0.49 (q = 0.67)	-0.6 (q = 0.73)	0.09 (q = 0.95)	-0.14 (q = 0.89)	0.6 (q = 0.69)	-0.31 (q = 0.9)	0.03 (q = 1)
Atopobium	-0.83 (q = 0.72)	0.03 (q = 1)	0.49 (q = 0.67)	0.26 (q = 0.88)	0.89 (q = 0.8)	0.6 (q = 0.89)	0.37 (q = 0.76)	-0.77 (q = 0.8)	-0.26 (q = 0.88)
Collinsella	-0.6 (q = 0.9)	-0.09 (q = 0.98)	-0.2 (q = 0.88)	0.03 (q = 1)	0.43 (q = 0.93)	-0.31 (q = 0.89)	0.26 (q = 0.87)	-0.31 (q = 0.9)	-0.49 (q = 0.86)
Coriobacterium	-0.75 (q = 0.72)	-0.17 (q = 0.91)	0.32 (q = 0.77)	0.49 (q = 0.73)	0.9 (q = 0.8)	0.12 (q = 0.9)	0.32 (q = 0.79)	-0.84 (q = 0.8)	-0.32 (q = 0.88)
Slackia	-0.2 (q = 0.9)	-0.2 (q = 0.91)	0.54 (q = 0.67)	0.09 (q = 0.95)	0.49 (q = 0.93)	0.77 (q = 0.72)	0.14 (q = 0.87)	-0.6 (q = 0.8)	0.6 (q = 0.86)
unclass. Solirubrobacterales	0.39 (q = 0.9)	-0.13 (q = 0.91)	0.39 (q = 0.67)	-0.13 (q = 0.88)	-0.39 (q = 0.93)	0.65 (q = 0.72)	-0.39 (q = 0.7)	0.39 (q = 0.8)	0.65 (q = 0.69)
unclass. Patulibacteraceae	0.39 (q = 0.9)	-0.13 (q = 0.91)	0.39 (q = 0.67)	-0.13 (q = 0.88)	-0.39 (q = 0.93)	0.65 (q = 0.72)	-0.39 (q = 0.7)	0.39 (q = 0.8)	0.65 (q = 0.69)
unclass. Bacteroidales	-0.78 (q = 0.72)	-0.51 (q = 0.7)	0.68 (q = 0.67)	0.68 (q = 0.57)	0.85 (q = 0.8)	0.44 (q = 0.89)	-0.03 (q = 0.97)	-0.68 (q = 0.8)	-0.07 (q = 0.95)
Bacteroides	0.49 (q = 0.9)	-0.43 (q = 0.7)	0.14 (q = 0.88)	0.26 (q = 0.88)	-0.71 (q = 0.8)	-0.14 (q = 0.89)	-0.83 (q = 0.52)	0.89 (q = 0.8)	0.2 (q = 0.88)
Parabacteroides	-0.03 (q = 1)	0.14 (q = 0.91)	-0.31 (q = 0.78)	-0.09 (q = 0.95)	-0.37 (q = 0.94)	-0.43 (q = 0.89)	-0.2 (q = 0.87)	0.6 (q = 0.8)	-0.6 (q = 0.86)
Porphyromonas	-0.54 (q = 0.9)	0.44 (q = 0.7)	-0.51 (q = 0.67)	-0.51 (q = 0.73)	0.14 (q = 0.94)	-0.27 (q = 0.89)	0.44 (q = 0.7)	0.03 (q = 0.98)	-0.78 (q = 0.69)
unclass. Prevotellaceae	-0.65 (q = 0.72)	0.13 (q = 0.91)	0.13 (q = 0.88)	0.13 (q = 0.88)	0.39 (q = 0.93)	0.13 (q = 0.89)	0.13 (q = 0.87)	-0.13 (q = 0.9)	-0.65 (q = 0.69)
Prevotella	0.94 (q = 0.72)	0.09 (q = 0.98)	-0.31 (q = 0.78)	-0.2 (q = 0.88)	-0.94 (q = 0.8)	-0.37 (q = 0.89)	-0.43 (q = 0.7)	0.83 (q = 0.8)	0.31 (q = 0.88)

Table S9 continued.

**Genus**	**fruit intake**	**intake of mature leaves**	**intake of young leaves**	**intake of flowers**	**intake of TNCE**	**intake of crude protein**	**intake of fiber**	**intake of fat**	**overall energy intake**
unclass. Bacteroidales RF16	-0.65 (q = 0.72)	0.13 (q = 0.91)	0.13 (q = 0.88)	0.13 (q = 0.88)	0.39 (q = 0.93)	0.13 (q = 0.89)	0.13 (q = 0.87)	-0.13 (q = 0.9)	-0.65 (q = 0.69)
unclass. Bacteroidales S24-7	0.1 (q = 0.93)	-0.49 (q = 0.7)	-0.1 (q = 0.91)	0.29 (q = 0.88)	-0.1 (q = 0.95)	-0.49 (q = 0.89)	-0.29 (q = 0.82)	0.1 (q = 0.92)	0.1 (q = 0.92)
unclass. [Paraprevotellaceae]	-0.13 (q = 0.9)	0.39 (q = 0.7)	-0.65 (q = 0.67)	-0.65 (q = 0.57)	-0.13 (q = 0.94)	-0.39 (q = 0.89)	0.39 (q = 0.7)	0.13 (q = 0.9)	-0.39 (q = 0.86)
unclass. [Paraprevotellaceae] YRC22	-0.72 (q = 0.72)	-0.49 (q = 0.7)	0.75 (q = 0.67)	0.46 (q = 0.73)	0.7 (q = 0.8)	0.72 (q = 0.72)	-0.12 (q = 0.88)	-0.49 (q = 0.8)	0.12 (q = 0.9)
[Prevotella]	-0.65 (q = 0.72)	0.13 (q = 0.91)	0.13 (q = 0.88)	0.13 (q = 0.88)	0.39 (q = 0.93)	0.13 (q = 0.89)	0.13 (q = 0.87)	-0.13 (q = 0.9)	-0.65 (q = 0.69)
Flavobacterium	0.13 (q = 0.9)	0.65 (q = 0.51)	-0.39 (q = 0.67)	-0.39 (q = 0.73)	0.13 (q = 0.94)	-0.13 (q = 0.89)	0.65 (q = 0.52)	-0.39 (q = 0.8)	-0.13 (q = 0.88)
unclass. [Weeksellaceae]	-0.39 (q = 0.9)	-0.65 (q = 0.51)	0.65 (q = 0.67)	0.65 (q = 0.57)	0.65 (q = 0.8)	0.39 (q = 0.89)	-0.13 (q = 0.87)	-0.65 (q = 0.8)	0.39 (q = 0.86)
Wautersiella	-0.65 (q = 0.72)	0.13 (q = 0.91)	0.13 (q = 0.88)	0.13 (q = 0.88)	0.39 (q = 0.93)	0.13 (q = 0.89)	0.13 (q = 0.87)	-0.13 (q = 0.9)	-0.65 (q = 0.69)
unclass. Sphingobacteriaceae	0.13 (q = 0.9)	0.65 (q = 0.51)	-0.39 (q = 0.67)	-0.39 (q = 0.73)	0.13 (q = 0.94)	-0.13 (q = 0.89)	0.65 (q = 0.52)	-0.39 (q = 0.8)	-0.13 (q = 0.88)
Pedobacter	0.13 (q = 0.9)	0.65 (q = 0.51)	-0.39 (q = 0.67)	-0.39 (q = 0.73)	0.13 (q = 0.94)	-0.13 (q = 0.89)	0.65 (q = 0.52)	-0.39 (q = 0.8)	-0.13 (q = 0.88)
unclass. Dolo_23	0.13 (q = 0.9)	0.65 (q = 0.51)	-0.39 (q = 0.67)	-0.39 (q = 0.73)	0.13 (q = 0.94)	-0.13 (q = 0.89)	0.65 (q = 0.52)	-0.39 (q = 0.8)	-0.13 (q = 0.88)
unclass. Thermomicrobia JG30-KF-CM45	-0.13 (q = 0.9)	0.39 (q = 0.7)	-0.65 (q = 0.67)	-0.65 (q = 0.57)	-0.13 (q = 0.94)	-0.39 (q = 0.89)	0.39 (q = 0.7)	0.13 (q = 0.9)	-0.39 (q = 0.86)
unclass. Cyanobacteria 4C0d-2 YS2	0.37 (q = 0.9)	0.26 (q = 0.91)	-0.49 (q = 0.67)	-0.6 (q = 0.73)	-0.71 (q = 0.8)	-0.26 (q = 0.89)	-0.14 (q = 0.87)	0.77 (q = 0.8)	-0.09 (q = 0.95)
unclass. Streptophyta	0.37 (q = 0.9)	0.03 (q = 1)	0.31 (q = 0.78)	0.09 (q = 0.95)	0.03 (q = 1)	0.43 (q = 0.89)	0.03 (q = 1)	-0.26 (q = 0.9)	0.6 (q = 0.86)

Table S9 continued.

Genus	fruit intake	intake of mature leaves	intake of young leaves	intake of flowers	intake of TNCE	intake of crude protein	intake of fiber	intake of fat	overall energy intake
unclass. Elusimicrobiaceae	0.65 (q = 0.72)	-0.39 (q = 0.7)	-0.13 (q = 0.88)	0.39 (q = 0.73)	-0.65 (q = 0.8)	-0.65 (q = 0.72)	-0.65 (q = 0.52)	0.65 (q = 0.8)	0.13 (q = 0.88)
_Alicyclobacillus	-0.85 (q = 0.72)	-0.3 (q = 0.87)	0.54 (q = 0.67)	0.54 (q = 0.73)	0.78 (q = 0.8)	0.37 (q = 0.89)	0.03 (q = 0.97)	-0.54 (q = 0.8)	-0.34 (q = 0.88)
unclass. Planococcaceae	-0.65 (q = 0.72)	0.13 (q = 0.91)	0.13 (q = 0.88)	0.13 (q = 0.88)	0.39 (q = 0.93)	0.13 (q = 0.89)	0.13 (q = 0.87)	-0.13 (q = 0.9)	-0.65 (q = 0.69)
Kurthia	-0.13 (q = 0.9)	0.39 (q = 0.7)	-0.65 (q = 0.67)	-0.65 (q = 0.57)	-0.13 (q = 0.94)	-0.39 (q = 0.89)	0.39 (q = 0.7)	0.13 (q = 0.9)	-0.39 (q = 0.86)
Staphylococcus	-0.39 (q = 0.9)	-0.65 (q = 0.51)	0.65 (q = 0.67)	0.65 (q = 0.57)	0.65 (q = 0.8)	0.39 (q = 0.89)	-0.13 (q = 0.87)	-0.65 (q = 0.8)	0.39 (q = 0.86)
unclass. Aerococcaceae	-0.13 (q = 0.9)	0.39 (q = 0.7)	-0.65 (q = 0.67)	-0.65 (q = 0.57)	-0.13 (q = 0.94)	-0.39 (q = 0.89)	0.39 (q = 0.7)	0.13 (q = 0.9)	-0.39 (q = 0.86)
Aerococcus	0.13 (q = 0.9)	0.65 (q = 0.51)	-0.39 (q = 0.67)	-0.39 (q = 0.73)	0.13 (q = 0.94)	-0.13 (q = 0.89)	0.65 (q = 0.52)	-0.39 (q = 0.8)	-0.13 (q = 0.88)
Facklamia	0.52 (q = 0.9)	0.52 (q = 0.7)	-0.76 (q = 0.67)	-0.39 (q = 0.73)	-0.39 (q = 0.93)	-0.76 (q = 0.72)	0.33 (q = 0.76)	0.15 (q = 0.9)	-0.21 (q = 0.88)
Desemzia	-0.13 (q = 0.9)	0.39 (q = 0.7)	-0.65 (q = 0.67)	-0.65 (q = 0.57)	-0.13 (q = 0.94)	-0.39 (q = 0.89)	0.39 (q = 0.7)	0.13 (q = 0.9)	-0.39 (q = 0.86)
Enterococcus	0.13 (q = 0.9)	0.65 (q = 0.51)	-0.39 (q = 0.67)	-0.39 (q = 0.73)	0.13 (q = 0.94)	-0.13 (q = 0.89)	0.65 (q = 0.52)	-0.39 (q = 0.8)	-0.13 (q = 0.88)
Lactobacillus	0.13 (q = 0.9)	0.65 (q = 0.51)	-0.39 (q = 0.67)	-0.39 (q = 0.73)	0.13 (q = 0.94)	-0.13 (q = 0.89)	0.65 (q = 0.52)	-0.39 (q = 0.8)	-0.13 (q = 0.88)
Streptococcus	-0.09 (q = 0.96)	0.89 (q = 0.51)	-0.54 (q = 0.67)	-0.89 (q = 0.57)	0.09 (q = 0.95)	0.09 (q = 0.96)	0.83 (q = 0.52)	-0.26 (q = 0.9)	-0.26 (q = 0.88)
Turicibacter	0.13 (q = 0.9)	0.65 (q = 0.51)	-0.39 (q = 0.67)	-0.39 (q = 0.73)	0.13 (q = 0.94)	-0.13 (q = 0.89)	0.65 (q = 0.52)	-0.39 (q = 0.8)	-0.13 (q = 0.88)
unclass. Clostridiales	-0.94 (q = 0.72)	0.14 (q = 0.91)	0.26 (q = 0.88)	0.14 (q = 0.88)	0.94 (q = 0.8)	0.37 (q = 0.89)	0.54 (q = 0.7)	-0.83 (q = 0.8)	-0.49 (q = 0.86)

Table S9 continued.

Genus	fruit intake	intake of mature leaves	intake of young leaves	intake of flowers	intake of TNCE	intake of crude protein	intake of fiber	intake of fat	overall energy intake
unclass. Clostridiaceae	-0.06 (q = 0.96)	0.81 (q = 0.51)	-0.49 (q = 0.67)	-0.84 (q = 0.57)	0.12 (q = 0.95)	0.12 (q = 0.9)	0.81 (q = 0.52)	-0.32 (q = 0.88)	-0.14 (q = 0.88)
Candidatus Arthromitus	0.13 (q = 0.9)	0.65 (q = 0.51)	-0.39 (q = 0.67)	-0.39 (q = 0.73)	0.13 (q = 0.94)	-0.13 (q = 0.89)	0.65 (q = 0.52)	-0.39 (q = 0.8)	-0.13 (q = 0.88)
Clostridium	-0.12 (q = 0.91)	0.49 (q = 0.7)	-0.46 (q = 0.67)	-0.64 (q = 0.61)	0.2 (q = 0.94)	-0.06 (q = 0.96)	0.7 (q = 0.52)	-0.41 (q = 0.8)	-0.06 (q = 0.95)
Sarcina	-0.13 (q = 0.9)	0.39 (q = 0.7)	-0.65 (q = 0.67)	-0.65 (q = 0.57)	-0.13 (q = 0.94)	-0.39 (q = 0.89)	0.39 (q = 0.7)	0.13 (q = 0.9)	-0.39 (q = 0.86)
Anaerofustis	-0.66 (q = 0.72)	0.6 (q = 0.69)	-0.6 (q = 0.67)	-0.49 (q = 0.73)	0.43 (q = 0.93)	-0.37 (q = 0.89)	0.77 (q = 0.52)	-0.37 (q = 0.85)	-0.89 (q = 0.69)
unclass. Lachnospiraceae	-0.03 (q = 1)	0.71 (q = 0.51)	-0.43 (q = 0.67)	-0.77 (q = 0.57)	0.14 (q = 0.94)	0.14 (q = 0.89)	0.77 (q = 0.52)	-0.37 (q = 0.85)	-0.03 (q = 1)
Anaerostipes	-0.77 (q = 0.72)	-0.37 (q = 0.79)	0.31 (q = 0.78)	0.43 (q = 0.73)	0.54 (q = 0.93)	0.09 (q = 0.96)	-0.09 (q = 0.95)	-0.26 (q = 0.9)	-0.43 (q = 0.86)
Blautia	-0.66 (q = 0.72)	-0.03 (q = 1)	-0.03 (q = 1)	-0.14 (q = 0.88)	0.31 (q = 0.94)	0.09 (q = 0.96)	0.14 (q = 0.87)	-0.09 (q = 0.96)	-0.43 (q = 0.86)
Butyrivibrio	-0.31 (q = 0.9)	0.77 (q = 0.51)	-0.2 (q = 0.88)	-0.54 (q = 0.73)	0.31 (q = 0.94)	0.31 (q = 0.89)	0.71 (q = 0.52)	-0.37 (q = 0.85)	-0.37 (q = 0.88)
Clostridium	-0.31 (q = 0.9)	0.49 (q = 0.7)	-0.43 (q = 0.67)	-0.09 (q = 0.95)	0.2 (q = 0.94)	-0.49 (q = 0.89)	0.43 (q = 0.7)	-0.14 (q = 0.9)	-0.83 (q = 0.69)
Coprococcus	0.09 (q = 0.96)	-0.31 (q = 0.87)	0.49 (q = 0.67)	0.14 (q = 0.88)	0.26 (q = 0.94)	0.6 (q = 0.89)	-0.03 (q = 1)	-0.43 (q = 0.8)	0.77 (q = 0.69)
Dorea	-0.64 (q = 0.72)	0.46 (q = 0.7)	-0.03 (q = 0.97)	-0.14 (q = 0.88)	0.81 (q = 0.8)	0.23 (q = 0.89)	0.81 (q = 0.52)	-0.9 (q = 0.8)	-0.38 (q = 0.87)
Lachnospira	-0.94 (q = 0.72)	-0.09 (q = 0.98)	0.37 (q = 0.73)	0.14 (q = 0.88)	0.77 (q = 0.8)	0.49 (q = 0.89)	0.26 (q = 0.87)	-0.54 (q = 0.8)	-0.37 (q = 0.88)
Oribacterium	-0.5 (q = 0.9)	-0.68 (q = 0.51)	0.88 (q = 0.67)	0.77 (q = 0.57)	0.5 (q = 0.93)	0.62 (q = 0.81)	-0.44 (q = 0.7)	-0.26 (q = 0.9)	0.18 (q = 0.88)

Table S9 continued.

Genus	fruit intake	intake of mature leaves	intake of young leaves	intake of flowers	intake of TNCE	intake of crude protein	intake of fiber	intake of fat	overall energy intake
Roseburia	0.26 (q = 0.9)	0.6 (q = 0.69)	-0.49 (q = 0.67)	-0.71 (q = 0.57)	-0.09 (q = 0.95)	-0.03 (q = 1)	0.6 (q = 0.69)	-0.2 (q = 0.9)	0.14 (q = 0.88)
[Ruminococcus]	-0.2 (q = 0.9)	-0.03 (q = 1)	0.26 (q = 0.88)	-0.31 (q = 0.88)	0.09 (q = 0.95)	0.66 (q = 0.76)	0.03 (q = 1)	-0.03 (q = 1)	0.31 (q = 0.88)
Peptococcus	-0.39 (q = 0.9)	-0.65 (q = 0.51)	0.65 (q = 0.67)	0.65 (q = 0.57)	0.65 (q = 0.8)	0.39 (q = 0.89)	-0.13 (q = 0.87)	-0.65 (q = 0.8)	0.39 (q = 0.86)
[Clostridium]	-0.13 (q = 0.9)	0.39 (q = 0.7)	-0.65 (q = 0.67)	-0.65 (q = 0.57)	-0.13 (q = 0.94)	-0.39 (q = 0.89)	0.39 (q = 0.7)	0.13 (q = 0.9)	-0.39 (q = 0.86)
unclass. Ruminococcaceae	0.37 (q = 0.9)	0.49 (q = 0.7)	-0.26 (q = 0.88)	-0.6 (q = 0.73)	-0.14 (q = 0.94)	0.2 (q = 0.89)	0.43 (q = 0.7)	-0.14 (q = 0.9)	0.37 (q = 0.88)
Anaerofilum	-0.31 (q = 0.9)	-0.26 (q = 0.91)	0.71 (q = 0.67)	0.26 (q = 0.88)	0.6 (q = 0.93)	0.89 (q = 0.72)	0.09 (q = 0.95)	-0.66 (q = 0.8)	0.54 (q = 0.86)
Faecalibacterium	-0.25 (q = 0.9)	-0.43 (q = 0.7)	0.83 (q = 0.67)	0.34 (q = 0.82)	0.25 (q = 0.94)	0.93 (q = 0.72)	-0.37 (q = 0.74)	-0.09 (q = 0.92)	0.46 (q = 0.86)
Oscillospira	-0.71 (q = 0.72)	0.03 (q = 1)	0.03 (q = 1)	-0.2 (q = 0.88)	0.54 (q = 0.93)	0.26 (q = 0.89)	0.37 (q = 0.76)	-0.43 (q = 0.8)	-0.26 (q = 0.88)
Papillibacter	0.31 (q = 0.9)	0.6 (q = 0.69)	-0.2 (q = 0.88)	-0.43 (q = 0.73)	-0.26 (q = 0.94)	0.14 (q = 0.89)	0.26 (q = 0.87)	0.14 (q = 0.9)	-0.03 (q = 1)
Ruminococcus	-0.6 (q = 0.9)	-0.31 (q = 0.87)	0.71 (q = 0.67)	0.6 (q = 0.73)	0.83 (q = 0.8)	0.6 (q = 0.89)	0.09 (q = 0.95)	-0.77 (q = 0.8)	0.09 (q = 0.95)
unclass. Veillonellaceae	0.31 (q = 0.9)	-0.94 (q = 0.51)	0.6 (q = 0.67)	0.83 (q = 0.57)	-0.26 (q = 0.94)	0.03 (q = 1)	-0.94 (q = 0.52)	0.37 (q = 0.85)	0.54 (q = 0.86)
Anaerovibrio	-0.27 (q = 0.9)	-0.17 (q = 0.91)	0.34 (q = 0.74)	0.34 (q = 0.82)	0.68 (q = 0.8)	0.27 (q = 0.89)	0.3 (q = 0.8)	-0.85 (q = 0.8)	0.27 (q = 0.88)
Dialister	0.74 (q = 0.72)	-0.62 (q = 0.59)	0.18 (q = 0.88)	0.29 (q = 0.88)	-0.74 (q = 0.8)	-0.15 (q = 0.89)	-0.85 (q = 0.52)	0.74 (q = 0.8)	0.65 (q = 0.69)
Phascolarctobacterium	0.31 (q = 0.9)	-0.94 (q = 0.51)	0.6 (q = 0.67)	0.83 (q = 0.57)	-0.26 (q = 0.94)	0.03 (q = 1)	-0.94 (q = 0.52)	0.37 (q = 0.85)	0.54 (q = 0.86)

Table S9 continued.

**Genus**	**fruit intake**	**intake of mature leaves**	**intake of young leaves**	**intake of flowers**	**intake of TNCE**	**intake of crude protein**	**intake of fiber**	**intake of fat**	**overall energy intake**
unclass.[Mogibacteriaceae]	0 (q = 1)	0.55 (q = 0.7)	-0.58 (q = 0.67)	-0.17 (q = 0.88)	0.03 (q = 0.97)	-0.64 (q = 0.76)	0.46 (q = 0.7)	-0.12 (q = 0.91)	-0.64 (q = 0.69)
Anaerovorax	-0.35 (q = 0.9)	0.17 (q = 0.91)	-0.03 (q = 0.97)	-0.49 (q = 0.73)	0.12 (q = 0.95)	0.41 (q = 0.89)	0.23 (q = 0.87)	-0.03 (q = 0.98)	-0.03 (q = 0.97)
Mogibacterium	0.26 (q = 0.9)	0.6 (q = 0.69)	-0.49 (q = 0.67)	-0.71 (q = 0.57)	-0.09 (q = 0.95)	-0.03 (q = 1)	0.6 (q = 0.69)	-0.2 (q = 0.9)	0.14 (q = 0.88)
Anaerococcus	0.39 (q = 0.9)	-0.13 (q = 0.91)	0.39 (q = 0.67)	-0.13 (q = 0.88)	-0.39 (q = 0.93)	0.65 (q = 0.72)	-0.39 (q = 0.7)	0.39 (q = 0.8)	0.65 (q = 0.69)
GW-34	-0.13 (q = 0.9)	0.39 (q = 0.7)	-0.65 (q = 0.67)	-0.65 (q = 0.57)	-0.13 (q = 0.94)	-0.39 (q = 0.89)	0.39 (q = 0.7)	0.13 (q = 0.9)	-0.39 (q = 0.86)
Coprobacillus	-0.89 (q = 0.72)	0.26 (q = 0.91)	-0.2 (q = 0.88)	-0.09 (q = 0.95)	0.66 (q = 0.82)	-0.14 (q = 0.89)	0.54 (q = 0.7)	-0.49 (q = 0.8)	-0.83 (q = 0.69)
RFN20	0.15 (q = 0.9)	0.76 (q = 0.51)	-0.7 (q = 0.67)	-0.94 (q = 0.57)	-0.21 (q = 0.94)	-0.15 (q = 0.89)	0.64 (q = 0.56)	0.03 (q = 0.98)	-0.15 (q = 0.88)
[Eubacterium]	-0.13 (q = 0.9)	0.39 (q = 0.7)	-0.65 (q = 0.67)	-0.65 (q = 0.57)	-0.13 (q = 0.94)	-0.39 (q = 0.89)	0.39 (q = 0.7)	0.13 (q = 0.9)	-0.39 (q = 0.86)
unclass. Caulobacteraceae	-0.3 (q = 0.9)	0.68 (q = 0.51)	-0.27 (q = 0.83)	-0.27 (q = 0.88)	0.37 (q = 0.94)	-0.03 (q = 0.99)	0.68 (q = 0.52)	-0.44 (q = 0.8)	-0.54 (q = 0.86)
unclass. Alphaproteobacteria RF32	0.13 (q = 0.9)	0.65 (q = 0.51)	-0.39 (q = 0.67)	-0.39 (q = 0.73)	0.13 (q = 0.94)	-0.13 (q = 0.89)	0.65 (q = 0.52)	-0.39 (q = 0.8)	-0.13 (q = 0.88)
unclass. Aurantimonadaceae	0.13 (q = 0.9)	0.65 (q = 0.51)	-0.39 (q = 0.67)	-0.39 (q = 0.73)	0.13 (q = 0.94)	-0.13 (q = 0.89)	0.65 (q = 0.52)	-0.39 (q = 0.8)	-0.13 (q = 0.88)
unclass. Bradyrhizobiaceae	0.65 (q = 0.72)	-0.39 (q = 0.7)	-0.13 (q = 0.88)	0.39 (q = 0.73)	-0.65 (q = 0.8)	-0.65 (q = 0.72)	-0.65 (q = 0.52)	0.65 (q = 0.8)	0.13 (q = 0.88)
Bosea	-0.65 (q = 0.72)	0.13 (q = 0.91)	0.13 (q = 0.88)	0.13 (q = 0.88)	0.39 (q = 0.93)	0.13 (q = 0.89)	0.13 (q = 0.87)	-0.13 (q = 0.9)	-0.65 (q = 0.69)
Devosia	-0.39 (q = 0.9)	-0.65 (q = 0.51)	0.65 (q = 0.67)	0.65 (q = 0.57)	0.65 (q = 0.8)	0.39 (q = 0.89)	-0.13 (q = 0.87)	-0.65 (q = 0.8)	0.39 (q = 0.86)

Table S9 continued.

Genus	fruit intake	intake of mature leaves	intake of young leaves	intake of flowers	intake of TNCE	intake of crude protein	intake of fiber	intake of fat	overall energy intake
unclass. Methylobacteriaceae	0.39 (q = 0.9)	-0.13 (q = 0.91)	0.39 (q = 0.67)	-0.13 (q = 0.88)	-0.39 (q = 0.93)	0.65 (q = 0.72)	-0.39 (q = 0.7)	0.39 (q = 0.8)	0.65 (q = 0.69)
Methylobacterium	0.37 (q = 0.9)	0.51 (q = 0.7)	-0.1 (q = 0.91)	-0.44 (q = 0.73)	-0.14 (q = 0.94)	0.3 (q = 0.89)	0.34 (q = 0.76)	-0.1 (q = 0.92)	0.3 (q = 0.88)
Rhizobium	0.65 (q = 0.72)	-0.39 (q = 0.7)	-0.13 (q = 0.88)	0.39 (q = 0.73)	-0.65 (q = 0.8)	-0.65 (q = 0.72)	-0.65 (q = 0.52)	0.65 (q = 0.8)	0.13 (q = 0.88)
unclass. Rhodobacteraceae	-0.13 (q = 0.9)	0.39 (q = 0.7)	-0.65 (q = 0.67)	-0.65 (q = 0.57)	-0.13 (q = 0.94)	-0.39 (q = 0.89)	0.39 (q = 0.7)	0.13 (q = 0.9)	-0.39 (q = 0.86)
unclass. Rhodospirillales	0.13 (q = 0.9)	0.65 (q = 0.51)	-0.39 (q = 0.67)	-0.39 (q = 0.73)	0.13 (q = 0.94)	-0.13 (q = 0.89)	0.65 (q = 0.52)	-0.39 (q = 0.8)	-0.13 (q = 0.88)
unclass. Rhodospirillaceae	0.13 (q = 0.9)	0.65 (q = 0.51)	-0.39 (q = 0.67)	-0.39 (q = 0.73)	0.13 (q = 0.94)	-0.13 (q = 0.89)	0.65 (q = 0.52)	-0.39 (q = 0.8)	-0.13 (q = 0.88)
Aristolochia	-0.39 (q = 0.9)	-0.65 (q = 0.51)	0.65 (q = 0.67)	0.65 (q = 0.57)	0.65 (q = 0.8)	0.39 (q = 0.89)	-0.13 (q = 0.87)	-0.65 (q = 0.8)	0.39 (q = 0.86)
Euptelea	-0.39 (q = 0.9)	-0.65 (q = 0.51)	0.65 (q = 0.67)	0.65 (q = 0.57)	0.65 (q = 0.8)	0.39 (q = 0.89)	-0.13 (q = 0.87)	-0.65 (q = 0.8)	0.39 (q = 0.86)
Lupinus	0.37 (q = 0.9)	0.51 (q = 0.7)	-0.1 (q = 0.91)	-0.44 (q = 0.73)	-0.14 (q = 0.94)	0.3 (q = 0.89)	0.34 (q = 0.76)	-0.1 (q = 0.92)	0.3 (q = 0.88)
Nelumbo	0.37 (q = 0.9)	0.51 (q = 0.7)	-0.1 (q = 0.91)	-0.44 (q = 0.73)	-0.14 (q = 0.94)	0.3 (q = 0.89)	0.34 (q = 0.76)	-0.1 (q = 0.92)	0.3 (q = 0.88)
Oenothera	-0.03 (q = 0.98)	-0.06 (q = 0.98)	0.4 (q = 0.67)	0.15 (q = 0.88)	0.46 (q = 0.93)	0.52 (q = 0.89)	0.25 (q = 0.87)	-0.68 (q = 0.8)	0.52 (q = 0.86)
Sarcandra	-0.39 (q = 0.9)	-0.65 (q = 0.51)	0.65 (q = 0.67)	0.65 (q = 0.57)	0.65 (q = 0.8)	0.39 (q = 0.89)	-0.13 (q = 0.87)	-0.65 (q = 0.8)	0.39 (q = 0.86)
unclass. Sphingomonadaceae	0.39 (q = 0.9)	-0.13 (q = 0.91)	0.39 (q = 0.67)	-0.13 (q = 0.88)	-0.39 (q = 0.93)	0.65 (q = 0.72)	-0.39 (q = 0.7)	0.39 (q = 0.8)	0.65 (q = 0.69)
Novosphingobium	-0.13 (q = 0.9)	0.39 (q = 0.7)	-0.65 (q = 0.67)	-0.65 (q = 0.57)	-0.13 (q = 0.94)	-0.39 (q = 0.89)	0.39 (q = 0.7)	0.13 (q = 0.9)	-0.39 (q = 0.86)

Table S9 continued.

Genus	fruit intake	intake of mature leaves	intake of young leaves	intake of flowers	intake of TNCE	intake of crude protein	intake of fiber	intake of fat	overall energy intake
Sphingomonas	0.76 (q = 0.72)	0.15 (q = 0.91)	0.03 (q = 0.97)	-0.21 (q = 0.88)	-0.58 (q = 0.93)	0.21 (q = 0.89)	-0.21 (q = 0.87)	0.39 (q = 0.8)	0.58 (q = 0.86)
unclass. Burkholderiales	-0.13 (q = 0.9)	0.39 (q = 0.7)	-0.65 (q = 0.67)	-0.65 (q = 0.57)	-0.13 (q = 0.94)	-0.39 (q = 0.89)	0.39 (q = 0.7)	0.13 (q = 0.9)	-0.39 (q = 0.86)
Sutterella	0.54 (q = 0.9)	-0.77 (q = 0.51)	0.54 (q = 0.67)	0.77 (q = 0.57)	-0.26 (q = 0.94)	0.03 (q = 1)	-0.77 (q = 0.52)	0.2 (q = 0.9)	0.71 (q = 0.69)
unclass. Comamonadaceae	-0.07 (q = 0.96)	-0.03 (q = 0.99)	0.44 (q = 0.67)	-0.03 (q = 0.96)	-0.1 (q = 0.95)	0.68 (q = 0.72)	-0.27 (q = 0.84)	0.27 (q = 0.9)	0.17 (q = 0.88)
Aquincola	0.65 (q = 0.72)	-0.39 (q = 0.7)	-0.13 (q = 0.88)	0.39 (q = 0.73)	-0.65 (q = 0.8)	-0.65 (q = 0.72)	-0.65 (q = 0.52)	0.65 (q = 0.8)	0.13 (q = 0.88)
Comamonas	0.39 (q = 0.9)	-0.13 (q = 0.91)	0.39 (q = 0.67)	-0.13 (q = 0.88)	-0.39 (q = 0.93)	0.65 (q = 0.72)	-0.39 (q = 0.7)	0.39 (q = 0.8)	0.65 (q = 0.69)
Hylemonella	-0.39 (q = 0.9)	-0.65 (q = 0.51)	0.65 (q = 0.67)	0.65 (q = 0.57)	0.65 (q = 0.8)	0.39 (q = 0.89)	-0.13 (q = 0.87)	-0.65 (q = 0.8)	0.39 (q = 0.86)
Ramlibacter	0.39 (q = 0.9)	-0.13 (q = 0.91)	0.39 (q = 0.67)	-0.13 (q = 0.88)	-0.39 (q = 0.93)	0.65 (q = 0.72)	-0.39 (q = 0.7)	0.39 (q = 0.8)	0.65 (q = 0.69)
Rubrivivax	0.41 (q = 0.9)	0 (q = 1)	-0.62 (q = 0.67)	-0.21 (q = 0.88)	-0.62 (q = 0.83)	-0.83 (q = 0.72)	-0.21 (q = 0.87)	0.62 (q = 0.8)	-0.21 (q = 0.88)
unclass. Oxalobacteraceae	0.09 (q = 0.94)	0.79 (q = 0.51)	-0.32 (q = 0.76)	-0.74 (q = 0.57)	-0.06 (q = 0.95)	0.26 (q = 0.89)	0.59 (q = 0.68)	-0.09 (q = 0.92)	-0.09 (q = 0.93)
Oxalobacter	-0.23 (q = 0.9)	-0.06 (q = 0.98)	-0.14 (q = 0.88)	0.03 (q = 0.96)	-0.2 (q = 0.94)	-0.29 (q = 0.89)	-0.26 (q = 0.85)	0.49 (q = 0.8)	-0.55 (q = 0.86)
unclass. Betaproteobacteria MND1	0.13 (q = 0.9)	0.65 (q = 0.51)	-0.39 (q = 0.67)	-0.39 (q = 0.73)	0.13 (q = 0.94)	-0.13 (q = 0.89)	0.65 (q = 0.52)	-0.39 (q = 0.8)	-0.13 (q = 0.88)
Petrobacter	-0.39 (q = 0.9)	-0.65 (q = 0.51)	0.65 (q = 0.67)	0.65 (q = 0.57)	0.65 (q = 0.8)	0.39 (q = 0.89)	-0.13 (q = 0.87)	-0.65 (q = 0.8)	0.39 (q = 0.86)
unclass. Desulfovibrionaceae	-0.03 (q = 1)	-0.71 (q = 0.51)	0.6 (q = 0.67)	0.37 (q = 0.81)	0.09 (q = 0.95)	0.49 (q = 0.89)	-0.49 (q = 0.7)	-0.03 (q = 1)	0.66 (q = 0.69)

Table S9 continued.

Genus	fruit intake	intake of mature leaves	intake of young leaves	intake of flowers	intake of TNCE	intake of crude protein	intake of fiber	intake of fat	overall energy intake
Bilophila	0.31 (q = 0.9)	-0.77 (q = 0.51)	0.6 (q = 0.67)	0.49 (q = 0.73)	-0.09 (q = 0.95)	0.37 (q = 0.89)	-0.6 (q = 0.69)	0.03 (q = 1)	0.89 (q = 0.69)
Desulfovibrio	0.89 (q = 0.72)	-0.26 (q = 0.91)	0.2 (q = 0.88)	0.09 (q = 0.95)	-0.66 (q = 0.82)	0.14 (q = 0.89)	-0.54 (q = 0.7)	0.49 (q = 0.8)	0.83 (q = 0.69)
unclass. Deltaproteobacteria GMD14H09	-0.65 (q = 0.72)	0.13 (q = 0.91)	0.13 (q = 0.88)	0.13 (q = 0.88)	0.39 (q = 0.93)	0.13 (q = 0.89)	0.13 (q = 0.87)	-0.13 (q = 0.9)	-0.65 (q = 0.69)
unclas.Myxococcales	0.37 (q = 0.9)	0.51 (q = 0.7)	-0.1 (q = 0.91)	-0.44 (q = 0.73)	-0.14 (q = 0.94)	0.3 (q = 0.89)	0.34 (q = 0.76)	-0.1 (q = 0.92)	0.3 (q = 0.88)
unclass. Polyangiaceae	-0.13 (q = 0.9)	0.39 (q = 0.7)	-0.65 (q = 0.67)	-0.65 (q = 0.57)	-0.13 (q = 0.94)	-0.39 (q = 0.89)	0.39 (q = 0.7)	0.13 (q = 0.9)	-0.39 (q = 0.86)
Campylobacter	-0.09 (q = 0.96)	0.89 (q = 0.51)	-0.54 (q = 0.67)	-0.89 (q = 0.57)	0.09 (q = 0.95)	0.09 (q = 0.96)	0.83 (q = 0.52)	-0.26 (q = 0.9)	-0.26 (q = 0.88)
Flexispira	-0.77 (q = 0.72)	0.54 (q = 0.7)	-0.43 (q = 0.67)	-0.31 (q = 0.88)	0.54 (q = 0.93)	-0.26 (q = 0.89)	0.71 (q = 0.52)	-0.43 (q = 0.8)	-0.94 (q = 0.69)
unclass. Aeromonadaceae	-0.13 (q = 0.9)	0.39 (q = 0.7)	-0.65 (q = 0.67)	-0.65 (q = 0.57)	-0.13 (q = 0.94)	-0.39 (q = 0.89)	0.39 (q = 0.7)	0.13 (q = 0.9)	-0.39 (q = 0.86)
Anaerobiospirillum	0.39 (q = 0.9)	-0.13 (q = 0.91)	0.39 (q = 0.67)	-0.13 (q = 0.88)	-0.39 (q = 0.93)	0.65 (q = 0.72)	-0.39 (q = 0.7)	0.39 (q = 0.8)	0.65 (q = 0.69)
Succinivibrio	0.12 (q = 0.91)	0.46 (q = 0.7)	-0.37 (q = 0.69)	-0.06 (q = 0.95)	-0.03 (q = 0.97)	-0.43 (q = 0.89)	0.31 (q = 0.8)	-0.06 (q = 0.96)	-0.43 (q = 0.86)
Cellvibrio	0.13 (q = 0.9)	0.65 (q = 0.51)	-0.39 (q = 0.67)	-0.39 (q = 0.73)	0.13 (q = 0.94)	-0.13 (q = 0.89)	0.65 (q = 0.52)	-0.39 (q = 0.8)	-0.13 (q = 0.88)
unclass. [Chromatiaceae]	-0.39 (q = 0.9)	-0.65 (q = 0.51)	0.65 (q = 0.67)	0.65 (q = 0.57)	0.65 (q = 0.8)	0.39 (q = 0.89)	-0.13 (q = 0.87)	-0.65 (q = 0.8)	0.39 (q = 0.86)
unclass. Enterobacteriaceae	-0.14 (q = 0.9)	0.94 (q = 0.51)	-0.83 (q = 0.67)	-0.94 (q = 0.57)	-0.09 (q = 0.95)	-0.31 (q = 0.89)	0.77 (q = 0.52)	0.03 (q = 1)	-0.66 (q = 0.69)
Citrobacter	-0.13 (q = 0.9)	0.39 (q = 0.7)	-0.65 (q = 0.67)	-0.65 (q = 0.57)	-0.13 (q = 0.94)	-0.39 (q = 0.89)	0.39 (q = 0.7)	0.13 (q = 0.9)	-0.39 (q = 0.86)
Erwinia	-0.07 (q = 0.96)	-0.03 (q = 0.99)	0.44 (q = 0.67)	-0.03 (q = 0.96)	-0.1 (q = 0.95)	0.68 (q = 0.72)	-0.27 (q = 0.84)	0.27 (q = 0.9)	0.17 (q = 0.88)

Table S9 continued.

Genus	fruit intake	intake of mature leaves	intake of young leaves	intake of flowers	intake of TNCE	intake of crude protein	intake of fiber	intake of fat	overall energy intake
Escherichia	0.03 (q = 0.98)	0.85 (q = 0.51)	-0.78 (q = 0.67)	-0.78 (q = 0.57)	0.03 (q = 0.97)	-0.37 (q = 0.89)	0.85 (q = 0.52)	-0.27 (q = 0.9)	-0.37 (q = 0.87)
Serratia	0.13 (q = 0.9)	0.65 (q = 0.51)	-0.39 (q = 0.67)	-0.39 (q = 0.73)	0.13 (q = 0.94)	-0.13 (q = 0.89)	0.65 (q = 0.52)	-0.39 (q = 0.8)	-0.13 (q = 0.88)
Shigella	-0.26 (q = 0.9)	0.93 (q = 0.51)	-0.75 (q = 0.67)	-0.93 (q = 0.57)	0.06 (q = 0.95)	-0.2 (q = 0.89)	0.84 (q = 0.52)	-0.12 (q = 0.91)	-0.64 (q = 0.69)
unclass. Pasteurellaceae	0.21 (q = 0.9)	0.44 (q = 0.7)	-0.09 (q = 0.92)	-0.5 (q = 0.73)	0.06 (q = 0.95)	0.38 (q = 0.89)	0.47 (q = 0.7)	-0.32 (q = 0.88)	0.38 (q = 0.87)
Actinobacillus	0.65 (q = 0.72)	-0.39 (q = 0.7)	-0.13 (q = 0.88)	0.39 (q = 0.73)	-0.65 (q = 0.8)	-0.65 (q = 0.72)	-0.65 (q = 0.52)	0.65 (q = 0.8)	0.13 (q = 0.88)
Aggregatibacter	0.68 (q = 0.72)	0.07 (q = 0.98)	-0.37 (q = 0.69)	0.1 (q = 0.92)	-0.51 (q = 0.93)	-0.68 (q = 0.72)	-0.17 (q = 0.87)	0.34 (q = 0.86)	0.03 (q = 0.97)
Haemophilus	-0.78 (q = 0.72)	-0.51 (q = 0.7)	0.68 (q = 0.67)	0.68 (q = 0.57)	0.85 (q = 0.8)	0.44 (q = 0.89)	-0.03 (q = 0.97)	-0.68 (q = 0.8)	-0.07 (q = 0.95)
Lonepinella	0.13 (q = 0.9)	0.65 (q = 0.51)	-0.39 (q = 0.67)	-0.39 (q = 0.73)	0.13 (q = 0.94)	-0.13 (q = 0.89)	0.65 (q = 0.52)	-0.39 (q = 0.8)	-0.13 (q = 0.88)
Mannheimia	0.75 (q = 0.72)	-0.12 (q = 0.93)	0.03 (q = 0.97)	0.26 (q = 0.88)	-0.52 (q = 0.93)	-0.23 (q = 0.89)	-0.41 (q = 0.7)	0.38 (q = 0.83)	0.38 (q = 0.87)
Pasteurella	0.7 (q = 0.72)	-0.76 (q = 0.51)	0.46 (q = 0.67)	0.58 (q = 0.73)	-0.58 (q = 0.93)	0.03 (q = 0.99)	-0.94 (q = 0.52)	0.58 (q = 0.8)	0.76 (q = 0.69)
Acinetobacter	-0.62 (q = 0.77)	0.62 (q = 0.59)	-0.62 (q = 0.67)	-0.44 (q = 0.73)	0.35 (q = 0.94)	-0.44 (q = 0.89)	0.71 (q = 0.52)	-0.26 (q = 0.9)	-0.97 (q = 0.22)
Pseudomonas	-0.37 (q = 0.9)	-0.07 (q = 0.98)	-0.17 (q = 0.88)	-0.17 (q = 0.88)	0.3 (q = 0.94)	-0.1 (q = 0.92)	0.27 (q = 0.84)	-0.3 (q = 0.9)	-0.1 (q = 0.92)
unclass. Sinobacteraceae	0.13 (q = 0.9)	0.65 (q = 0.51)	-0.39 (q = 0.67)	-0.39 (q = 0.73)	0.13 (q = 0.94)	-0.13 (q = 0.89)	0.65 (q = 0.52)	-0.39 (q = 0.8)	-0.13 (q = 0.88)
unclass. Xanthomonadaceae	0.13 (q = 0.9)	0.65 (q = 0.51)	-0.39 (q = 0.67)	-0.39 (q = 0.73)	0.13 (q = 0.94)	-0.13 (q = 0.89)	0.65 (q = 0.52)	-0.39 (q = 0.8)	-0.13 (q = 0.88)
Pseudoxanthomonas	-0.65 (q = 0.72)	0.13 (q = 0.91)	0.13 (q = 0.88)	0.13 (q = 0.88)	0.39 (q = 0.93)	0.13 (q = 0.89)	0.13 (q = 0.87)	-0.13 (q = 0.9)	-0.65 (q = 0.69)

Table S9 continued.

Genus	fruit intake	intake of mature leaves	intake of young leaves	intake of flowers	intake of TNCE	intake of crude protein	intake of fiber	intake of fat	overall energy intake
Stenotrophomonas	-0.13 (q = 0.9)	0.39 (q = 0.7)	-0.65 (q = 0.67)	-0.65 (q = 0.57)	-0.13 (q = 0.94)	-0.39 (q = 0.89)	0.39 (q = 0.7)	0.13 (q = 0.9)	-0.39 (q = 0.86)
Xanthomonas	0.65 (q = 0.72)	-0.39 (q = 0.7)	-0.13 (q = 0.88)	0.39 (q = 0.73)	-0.65 (q = 0.8)	-0.65 (q = 0.72)	-0.65 (q = 0.52)	0.65 (q = 0.8)	0.13 (q = 0.88)
Treponema	0.65 (q = 0.72)	-0.39 (q = 0.7)	-0.13 (q = 0.88)	0.39 (q = 0.73)	-0.65 (q = 0.8)	-0.65 (q = 0.72)	-0.65 (q = 0.52)	0.65 (q = 0.8)	0.13 (q = 0.88)
Synergistes	-0.13 (q = 0.9)	0.39 (q = 0.7)	-0.65 (q = 0.67)	-0.65 (q = 0.57)	-0.13 (q = 0.94)	-0.39 (q = 0.89)	0.39 (q = 0.7)	0.13 (q = 0.9)	-0.39 (q = 0.86)
unclass. Mollicutes	-0.89 (q = 0.72)	0.26 (q = 0.91)	-0.2 (q = 0.88)	-0.09 (q = 0.95)	0.66 (q = 0.82)	-0.14 (q = 0.89)	0.54 (q = 0.7)	-0.49 (q = 0.8)	-0.83 (q = 0.69)
unclass. RF3 ML615J-28	0.65 (q = 0.72)	-0.39 (q = 0.7)	-0.13 (q = 0.88)	0.39 (q = 0.73)	-0.65 (q = 0.8)	-0.65 (q = 0.72)	-0.65 (q = 0.52)	0.65 (q = 0.8)	0.13 (q = 0.88)
Opitutus	0.39 (q = 0.9)	-0.13 (q = 0.91)	0.39 (q = 0.67)	-0.13 (q = 0.88)	-0.39 (q = 0.93)	0.65 (q = 0.72)	-0.39 (q = 0.7)	0.39 (q = 0.8)	0.65 (q = 0.69)
unclass. [Cerasicoccaceae]	-0.26 (q = 0.9)	-0.6 (q = 0.69)	0.49 (q = 0.67)	0.71 (q = 0.57)	0.09 (q = 0.95)	0.03 (q = 1)	-0.6 (q = 0.69)	0.2 (q = 0.9)	-0.14 (q = 0.88)
unclass. RFP12	0.68 (q = 0.72)	0.07 (q = 0.98)	-0.37 (q = 0.69)	0.1 (q = 0.92)	-0.51 (q = 0.93)	-0.68 (q = 0.72)	-0.17 (q = 0.87)	0.34 (q = 0.86)	0.03 (q = 0.97)
unclass. WPS-2	-0.13 (q = 0.9)	0.39 (q = 0.7)	-0.65 (q = 0.67)	-0.65 (q = 0.57)	-0.13 (q = 0.94)	-0.39 (q = 0.89)	0.39 (q = 0.7)	0.13 (q = 0.9)	-0.39 (q = 0.86)
